# Supplementary material for: Fermionic parton theory of Rydberg ℤ2 quantum spin liquids
Source: Nat Commun. 2026 Jul 13;17:8588. doi: 10.1038/s41467-026-75245-9 (PMC13487239; doi:10.1038/s41467-026-75245-9)
Supplement: Supplementary file 1 [file 41467_2026_75245_MOESM1_ESM.pdf]

Supplementary Information for
"Fermionic parton theory of Rydberg $\mathbb{Z}_2$ quantum spin liquids"

Atanu Maity,[1,2] Yasir Iqbal,[2] and Rhine Samajdar[3,4]

[1] *Institut für Theoretische Physik und Astrophysik and Würzburg-Dresden Cluster of Excellence ct.qmat, Julius-Maximilians-Universität Würzburg, Am Hubland, Campus Süd, Würzburg 97074, Germany*
[2] *Department of Physics and Quantum Centre for Diamond and Emergent Materials (QuCenDiEM), Indian Institute of Technology Madras, Chennai 600036, India*
[3] *Department of Physics, Princeton University, Princeton, NJ 08544, USA*
[4] *Princeton Center for Theoretical Science, Princeton University, Princeton, NJ 08544, USA*

In this Supplementary Information, we provide further details on the projective symmetry group (PSG) classification on the ruby lattice and the mean-field decoupling of the Rydberg Hamiltonian in the fermionic parton language. We also analytically compute and showcase the static as well as dynamical spin structure factors for all the identified *Ansätze* over a broad range of mean-field parameters.

SI. SYMMETRIES AND PSG CLASSIFICATION

On the ruby lattice, the Rydberg Hamiltonian defined in Eq. (1) of the main text is explicitly invariant under the following symmetry transformations:

$$T_1 : (S_i^x, S_i^y, S_i^z) \to \left(S_{T_1(i)}^x, S_{T_1(i)}^y, S_{T_1(i)}^z\right), \qquad \text{(S1)}$$

$$T_2 : (S_i^x, S_i^y, S_i^z) \to \left(S_{T_2(i)}^x, S_{T_2(i)}^y, S_{T_2(i)}^z\right), \qquad \text{(S2)}$$

$$C_6 : (S_i^x, S_i^y, S_i^z) \to \left(S_{C_6(i)}^x, S_{C_6(i)}^y, S_{C_6(i)}^z\right), \qquad \text{(S3)}$$

$$R : (S_i^x, S_i^y, S_i^z) \to \left(S_{R(i)}^x, S_{R(i)}^y, S_{R(i)}^z\right), \qquad \text{(S4)}$$

$$\Theta : (S_i^x, S_i^y, S_i^z) \to (S_i^x, -S_i^y, S_i^z). \qquad \text{(S5)}$$

Here, $T_1(i)$ and $T_2(i)$ denote translations of the lattice coordinate i by the lattice vectors $\mathbf{T}_1$ and $\mathbf{T}_2$, respectively, $C_6(i)$ implements a sixfold rotation around the axis perpendicular to the plane, and $R(i)$ represents a reflection about the x-axis. Note that time reversal Θ does not act on the lattice coordinates.

In parton space, the linear actions of the space-group symmetries are straightforward since they simply modify the site indices. However, the action of Θ has to be defined more carefully. Under time reversal, we have $\Theta : \{f_{i\uparrow} \to -f_{i\downarrow}^\dagger, f_{i\downarrow} \to f_{i\uparrow}^\dagger\}$, or equivalently, on the spinor doublet, $\Theta : \psi_i \to \psi_i \, i\tau^y$. Accordingly, the spin operators transform as

$$S_i^\gamma = \frac{1}{2}\mathrm{Tr}\left[\psi_i^\dagger \tau^\gamma \psi_i\right] \to \frac{1}{2}\mathrm{Tr}\left[(-i\tau^y)\psi_i^\dagger (\tau^\gamma)^\star \psi_i \,(i\tau^y)\right] = \frac{1}{2}\mathrm{Tr}\left[\psi_i^\dagger (\tau^\gamma)^\star \psi_i\right] = (-1)^{\delta_{\gamma,y}} S_i^\gamma.$$

The projective extension of the symmetry group can be directly read off from Ref. 1 as:

$$W_{T_1}(x,y,s) = \eta^y \tau^0, \; W_{T_2}(x,y,s) = \tau^0, \qquad \text{(S6)}$$

$$W_{C_6}(x,y,s) = \eta^{xy + \frac{y}{2}(y+1)} \left(\eta_{C_6}\right)^{\delta_{s,6}} \tau^0, \qquad \text{(S7)}$$

$$W_R(x,y,s) = \eta^{\frac{x}{2}(x+1)} \left(\eta_{C_6}\right)^{\delta_{s,5}} \left(\eta_{C_6 R}\right)^{\delta_{\mathrm{mod}(s,2),0}} g_R, \qquad \text{(S8)}$$

$$W_\Theta(x,y,s) = \eta_{\Theta C_6}^s \, g_\Theta, \; g_R^2 = \eta_R \tau^0, \qquad \text{(S9)}$$

where (x, y, s) denotes the position of a site on sublattice s in the unit cell located at (x, y). In the equations above, all the parameters $\{\eta, \eta_{C_6}, \eta_R, \eta_{C_6 R}, \eta_{\Theta C_6}\}$ can take values ± 1 and the gauge-independent choices of the matrices g_R and g_Θ are listed in Supplementary Table 1 below. In total, we obtain 64 projective symmetry extensions for a $\mathbb{Z}_2$ IGG.

η_R	g_R	g_Θ	Set of η parameters	# of PSGs
$+1$	τ^0	$i\tau^y$	$\{\eta_{\Theta C_6}, \eta, \eta_{C_6}, \eta_{C_6 R}\}$	2^4
$+1$	τ^0	τ^0	$\{\eta_{\Theta C_6} = -1, \eta, \eta_{C_6}, \eta_{C_6 R}\}$	2^3
-1	$i\tau^z$	$i\tau^y$	$\{\eta_{\Theta C_6}, \eta, \eta_{C_6}, \eta_{C_6 R}\}$	2^4
-1	$i\tau^z$	$i\tau^z$	$\{\eta_{\Theta C_6}, \eta, \eta_{C_6}, \eta_{C_6 R}\}$	2^4
-1	$i\tau^z$	τ^0	$\{\eta_{\Theta C_6} = -1, \eta, \eta_{C_6}, \eta_{C_6 R}\}$	2^3

Supplementary Table 1. All possible gauge-inequivalent choices of the binary parameter η_R, and the two matrices g_R, g_Θ, which together define a total of 64 $\mathbb{Z}_2$ PSG solutions.

Once the PSGs have been specified, we can proceed to construct the mean-field *Ansätze*. In our projective construction, the symmetries of the mean fields are preserved as

$$\begin{aligned} u_{ij}^0 &= (-1)^{\delta_{\mathcal{O},\Theta}} W_{\mathcal{O}}^\dagger(\mathcal{O}(i)) u_{\mathcal{O}(i)\mathcal{O}(j)}^0 W_{\mathcal{O}}(\mathcal{O}(j)), \\ u_{ij}^{\gamma'} &= W_{\mathcal{O}}^\dagger(\mathcal{O}(i)) u_{\mathcal{O}(i)\mathcal{O}(j)}^\gamma \mathcal{R}_{\mathcal{O}}^{\gamma\gamma'} W_{\mathcal{O}}(\mathcal{O}(j)), \end{aligned} \qquad \text{(S10)}$$

where $\gamma, \gamma' = \{x, y, z\}$ and $\mathcal{R}_{\mathcal{O}}$ are 3×3 matrices. These matrices are, in general, nontrivial for real spin operators which transform as pseudovectors under symmetry operations. In our case, however, for the lattice space-group symmetry elements, $\mathcal{R}_{\mathcal{O}} = \mathbb{1}_{3\times 3}$.

Now, let us consider the effect of time reversal on the *Ansatz*. For singlet fields, i.e., $u_{ij}^0 = is_{ij}^0 \tau^0 + s_{ij}^1 \tau^x +$

$s^2_{ij}\tau^y + s^3_{ij}\tau^z$, we have

$$\Theta : \mathrm{Tr}\left[\psi_i\, u^0_{ij}\psi^\dagger_j\right] \to \mathrm{Tr}\left[\psi_i\,(i\tau^y)(u^0_{ij})^\star(-i\tau^y)\psi^\dagger_j\right] = -\,\mathrm{Tr}\left[\psi_i\, u^0_{ij}\psi^\dagger_j\right], \tag{S11}$$

whereas for the triplet components, i.e., $u^\gamma_{ij} = t^{\gamma,0}_{ij}\tau^0 + i(t^{\gamma,1}_{ij}\tau^x + t^{\gamma,2}_{ij}\tau^y + t^{\gamma,3}_{ij}\tau^z)$,

$$\Theta : \mathrm{Tr}\left[\tau^\gamma\psi_i\, u^\gamma_{ij}\psi^\dagger_j\right] \to \mathrm{Tr}\left[(\tau^\gamma)^\star\psi_i\,(i\tau^y)(u^\gamma_{ij})^\star(-i\tau^y)\psi^\dagger_j\right] = (-)^{\delta_{\gamma,y}}\,\mathrm{Tr}\left[\tau^\gamma\psi_i\, u^\gamma_{ij}\psi^\dagger_j\right]. \tag{S12}$$

Therefore, we obtain

$$\Theta : u^0_{ij} \to -u^0_{ij}, \tag{S13}$$

$$\Theta : \left\{u^x_{ij}, u^y_{ij}, u^z_{ij}\right\} \to \left\{u^x_{ij}, -u^y_{ij}, u^z_{ij}\right\}, \tag{S14}$$

so $\mathcal{R}_\Theta = \mathrm{Diag}[1,-1,1]$ with an additional negative sign for the singlet fields.

As mentioned in the main text, the full spatial structure of the *Ansatz* can be defined by specifying a few reference mean fields. Temporarily using the notation $u^\alpha_{s_1,s_2}$ ($s_1, s_2 = 1,\ldots,6$) to denote the directed link from a site on sublattice s_1 to a site on sublattice s_2, the sign structure of the various terms is given by

$$u^\alpha_{1,3} = u^\alpha_{2,4} = \eta u^\alpha_{3,5} = \eta\eta_{C_6}u^\alpha_{4,6} = \eta\eta_{C_6}u^\alpha_{5,1} = \eta u^\alpha_{6,2} \equiv \mathcal{U}^\alpha_1, \tag{S15}$$

$$u^\alpha_{1,2} = u^\alpha_{2,3} = u^\alpha_{3,4} = u^\alpha_{4,5} = \eta_{C_6}u^\alpha_{5,6} = u^\alpha_{6,1} \equiv \mathcal{U}^\alpha_2, \tag{S16}$$

$$u^\alpha_{1,4} = \eta\eta_{C_6}u^\alpha_{3,6} = \eta\eta_{C_6}u^\alpha_{5,2} \equiv \mathcal{U}^\alpha_3, \tag{S17}$$

$$u^\alpha_{2,5} = \eta\eta_{C_6}u^\alpha_{4,1} = \eta u^\alpha_{6,3} = \eta\eta_{C_6}\eta_{C_6R}g^\dagger_R\mathcal{U}^\alpha_3 g_R, \tag{S18}$$

where the subscript 1, 2, or 3 on $\mathcal{U}^\alpha$ indicates the first-, second-, or third-nearest-neighbor nature of the bond. Furthermore, the bonds connecting sites with different y-coordinates also change sign under translations by $\mathbf{T}_1$.

SII. MEAN-FIELD DECOUPLING

In the Abrikosov fermion representation $S^\gamma_i = \frac{1}{2}f^\dagger_{i\sigma}\tau^\gamma_{\sigma\sigma'}f_{i\sigma'}$, the Rydberg Hamiltonian can be recast as $H = H_0 + H_2 + H_4$, where

$$H_0 = -\frac{\Delta N}{2} + \sum_{\langle i,j\rangle}\frac{V_{ij}}{4}, \tag{S19}$$

$$H_2 = \frac{1}{2}\sum_i \Omega f^\dagger_{i,\sigma}\tau^x_{\sigma\sigma'}f_{i,\sigma'} - \frac{1}{2}\sum_i \Delta f^\dagger_{i,\sigma}\tau^z_{\sigma\sigma'}f_{i,\sigma'} + \sum_{\langle i,j\rangle}\frac{V_{ij}}{4}\left[f^\dagger_{i,\sigma}\tau^z_{\sigma\sigma'}f_{i,\sigma'} + f^\dagger_{j,\sigma}\tau^z_{\sigma\sigma'}f_{j,\sigma'}\right], \tag{S20}$$

$$H_4 = \frac{1}{4}\sum_{\langle i,j\rangle} V_{ij}\sigma\sigma' f^\dagger_{j\sigma'}f^\dagger_{i\sigma}f_{i\sigma}f_{j\sigma'}. \tag{S21}$$

Now, we perform a quadratic decoupling of the quartic term to obtain a mean-field theory as

$$\begin{aligned}
\sigma\sigma' f^\dagger_{j\sigma'}f^\dagger_{i\sigma}f_{i\sigma}f_{j\sigma'} = &\left\langle f^\dagger_{i\sigma}f^\dagger_{j\sigma}\right\rangle f_{i\sigma}f_{j\sigma} + \left\langle f_{i\sigma}f_{j\sigma}\right\rangle f^\dagger_{i\sigma}f^\dagger_{j\sigma} - \left\langle f^\dagger_{i\sigma}f^\dagger_{j\sigma}\right\rangle\left\langle f_{i\sigma}f_{j\sigma}\right\rangle \\
&- \left\langle f^\dagger_{i\sigma}f^\dagger_{j\bar\sigma}\right\rangle f_{i\sigma}f_{j\bar\sigma} - \left\langle f_{i\sigma}f_{j\bar\sigma}\right\rangle f^\dagger_{i\sigma}f^\dagger_{j\bar\sigma} + \left\langle f^\dagger_{i\sigma}f^\dagger_{j\bar\sigma}\right\rangle\left\langle f_{i\sigma}f_{j\bar\sigma}\right\rangle \\
&- \left\langle f^\dagger_{i\sigma}f_{j\sigma}\right\rangle f^\dagger_{j\sigma}f_{i\sigma} - \left\langle f^\dagger_{j\sigma}f_{i\sigma}\right\rangle f^\dagger_{i\sigma}f_{j\sigma} + \left\langle f^\dagger_{i\sigma}f_{j\sigma}\right\rangle\left\langle f^\dagger_{j\sigma}f_{i\sigma}\right\rangle \\
&+ \left\langle f^\dagger_{i\sigma}f_{j\bar\sigma}\right\rangle f^\dagger_{j\bar\sigma}f_{i\sigma} + \left\langle f^\dagger_{j\bar\sigma}f_{i\sigma}\right\rangle f^\dagger_{i\sigma}f_{j\bar\sigma} - \left\langle f^\dagger_{i\sigma}f_{j\bar\sigma}\right\rangle\left\langle f^\dagger_{j\bar\sigma}f_{i\sigma}\right\rangle \\
&+ \left\langle \sigma f^\dagger_{i\sigma}f_{i\sigma}\right\rangle \sigma' f^\dagger_{j\sigma'}f_{j\sigma'} + \left\langle \sigma' f^\dagger_{j\sigma'}f_{j\sigma'}\right\rangle \sigma f^\dagger_{i\sigma}f_{i\sigma} - \left\langle \sigma f^\dagger_{i\sigma}f_{i\sigma}\right\rangle\left\langle \sigma' f^\dagger_{j\sigma'}f_{j\sigma'}\right\rangle.
\end{aligned} \tag{S22}$$

Next, we define the following singlet and triplet hopping and pairing fields:

$$\chi_{ij} = f^\dagger_{i\uparrow}f_{j\uparrow} + f^\dagger_{i\downarrow}f_{j\downarrow}, \qquad \xi_{ij} = f^\dagger_{i\uparrow}f^\dagger_{j\downarrow} - f^\dagger_{i\downarrow}f^\dagger_{j\uparrow}, \tag{S23}$$

$$T^{x,h}_{ij} = f^\dagger_{i\uparrow}f_{j\downarrow} + f^\dagger_{i\downarrow}f_{j\uparrow}, \quad T^{y,h}_{ij} = -i(f^\dagger_{i\uparrow}f_{j\downarrow} - f^\dagger_{i\downarrow}f_{j\uparrow}), \quad T^{z,h}_{ij} = f^\dagger_{i\uparrow}f_{j\uparrow} - f^\dagger_{i\downarrow}f_{j\downarrow}, \tag{S24}$$

$$T^{x,p}_{ij} = f^\dagger_{i\downarrow}f^\dagger_{j\downarrow} - f^\dagger_{i\uparrow}f^\dagger_{j\uparrow}, \quad T^{y,p}_{ij} = i(f^\dagger_{i\downarrow}f^\dagger_{j\downarrow} + f^\dagger_{i\uparrow}f^\dagger_{j\uparrow}), \quad T^{z,p}_{ij} = f^\dagger_{i\uparrow}f^\dagger_{j\downarrow} + f^\dagger_{i\downarrow}f^\dagger_{j\uparrow}. \tag{S25}$$

In the mean-field approximation, i.e., $\langle\chi_{ij}\rangle = \chi_{ij}$, $\langle\xi_{ij}\rangle = \xi_{ij}$, and $\langle T^{\gamma,r}_{ij}\rangle = T^{\gamma,r}_{ij}$ (with $r = h,p$), the Hamiltonian reads

$$H = H_{\bar 0} + H_2 + H_{\bar 2}, \tag{S26}$$

where

$$H_{\bar{0}} = H_0 + \frac{1}{8}\sum_{\langle i,j\rangle} V_{ij}\left[|\chi_{ij}|^2 + |\xi_{ij}|^2 + \frac{1}{8}\sum_{\gamma,r}\zeta_\gamma |T_{ij}^{\gamma,r}|^2 - 2T_{ii}^{z,h}T_{jj}^{z,h}\right], \tag{S27}$$

$$H_2 = \frac{1}{2}\sum_i \Omega\, T_{ii}^{x,h} - \frac{1}{2}\sum_i \Delta T_{ii}^{z,h} + \sum_{\langle i,j\rangle}\frac{V_{ij}}{4}\left[T_{ii}^{z,h} + T_{jj}^{z,h}\right], \tag{S28}$$

$$H_{\bar{2}} = -\frac{1}{8}\sum_{\langle i,j\rangle} V_{ij}\left[\left(\chi_{ij}^\star\chi_{ij} + \xi_{ij}^\star\xi_{ij} + \sum_{\gamma,r}\zeta_\gamma (T_{ij}^{\gamma,r})^\star T_{ij}^{\gamma,r} + \text{h.c.}\right) - 2T_{ii}^{z,h}T_{jj}^{z,h} - 2T_{jj}^{z,h}T_{ii}^{z,h}\right], \tag{S29}$$

with $\zeta_z = -\zeta_x = -\zeta_y = +1$. Now, using the doublet ψ_i and the matrices

$$u_{ij}^0 = \begin{bmatrix}\chi_{ij} & \xi_{ij}\\ \xi_{ij}^\star & -\chi_{ij}^\star\end{bmatrix}, \quad u_{ij}^\gamma = \begin{bmatrix} T_{ij}^{\gamma,h} & T_{ij}^{\gamma,p}\\ -(T_{ij}^{\gamma,p})^\star & (T_{ij}^{\gamma,h})^\star\end{bmatrix}, \tag{S30}$$

one can rewrite the quadratic part, i.e., $H_q = H_2 + H_{\bar{2}}$, in the form

$$H_q = \frac{1}{16}\sum_{\langle i,j\rangle} V_{ij}\left(\text{Tr}[\tau^\alpha\psi_i\, u_{ij}^\alpha\psi_j^\dagger + \text{h.c.}] + \text{Tr}[\tau^z\psi_i\, u_{ii}^z\psi_i^\dagger] + \text{Tr}[\tau^z\psi_j\, u_{jj}^z\psi_j^\dagger]\right) + \frac{1}{4}\sum_i\left(\Omega\,\text{Tr}[\tau^x\psi_i\,\psi_i^\dagger] - \Delta\,\text{Tr}[\tau^z\psi_i\,\psi_i^\dagger]\right), \tag{S31}$$

where u_{ii}^z is a 2×2 diagonal matrix with elements $\tilde{T}_{ii}^{z,h}$ and $(\tilde{T}_{ii}^{z,h})^\dagger$; $\tilde{T}_{ii}^{z,h} \equiv 2T_{ii}^{z,h} + 1$. If we also restrict the Hamiltonian to m^{th}-nearest-neighbor (mNN) terms and there are, say, N_p pNN sites per unit cell, then we have

$$\begin{aligned} H_q =& \frac{1}{16}\sum_{p=1}^{m}\sum_{\langle i,j\rangle_p} V_p\left(\text{Tr}[\tau^\alpha\psi_i\, u_{ij}^\alpha\psi_j^\dagger + \text{h.c.}] + N_p\,\text{Tr}[\tau^z\psi_i\, u_{ii}^z\psi_i^\dagger]\right) + \frac{1}{4}\sum_i\left(\Omega\,\text{Tr}[\tau^x\psi_i\,\psi_i^\dagger] - \Delta\text{Tr}[\tau^z\psi_i\,\psi_i^\dagger]\right)\\ =& \frac{1}{16}\sum_{p=1}^{m}\sum_{\langle i,j\rangle_p} V_p\left(\text{Tr}[\tau^\alpha\psi_i\, u_{ij}^\alpha\psi_j^\dagger + \text{h.c.}]\right) + \frac{1}{4}\sum_i\left(\Omega\,\text{Tr}[\tau^x\psi_i\,\psi_i^\dagger] + \text{Tr}[\tau^z\psi_i\,\tilde{u}_{ii}^z\psi_i^\dagger]\right), \end{aligned} \tag{S32}$$

where we use the shorthand $\tilde{u}_{ii}^z \equiv -\Delta\tau^0 + u_{ii}^z\sum_{p=1}^m N_pV_p/8$, and V_p is the van der Waals interaction between p^{th}-neighboring sites. Note that each u_{ij}^α consists of a hopping and a pairing field which are, in general, complex. Additionally, we denote $u_{ii}^x = \Omega\tau^0$, rename $\tilde{u}_{ij}^z$ as u_{ij}^z, and absorb the constants by rescaling the u fields. This sequence of steps then finally brings us to expression for the quadratic Hamiltonian in Eq. (2) of the main text:

$$H_q = \sum_{p=1}^{m}\sum_{\langle i,j\rangle_p} V_p\,\text{Tr}\left[\tau^\alpha\psi_i\, u_{ij}^\alpha\psi_j^\dagger + \text{h.c.}\right] + \sum_{i,\alpha=\{x,y,z\}}\text{Tr}\left[\tau^\alpha\psi_i\, u_{ii}^\alpha\psi_i^\dagger\right]. \tag{S33}$$

SIII. SPIN STRUCTURE FACTORS

In this section, we first outline the procedure to calculate the spin structure factors in the fermionic spinon representation. Considering only the longitudinal component, the dynamical structure factor is defined as

$$\mathcal{S}(\mathbf{q},\omega) = \int_{-\infty}^{+\infty}\frac{dt}{2\pi N^2}e^{i\omega t}\sum_{i,j}e^{i\mathbf{q}\cdot\mathbf{r}_{ij}}\left(\langle S_i^z(t)S_j^z(0)\rangle - \langle S_i^z(t)\rangle\langle S_j^z(0)\rangle\right) \equiv \mathcal{S}_{\text{tot}}(\mathbf{q},\omega) - \mathcal{S}_{\text{dis}}(\mathbf{q},\omega), \tag{S34}$$

where $\mathbf{r}_{ij} \equiv \mathbf{r}_i - \mathbf{r}_j$ and N is the total number of spins. In this definition, the connected correlator $\mathcal{S}(\mathbf{q},\omega)$ is interpreted as the sum of two pieces: $\mathcal{S}_{\text{tot}}(\mathbf{q},\omega)$, which is the "total" correlation function $\langle S_i^z(t)S_j^z(0)\rangle$, and a disconnected part, $\mathcal{S}_{\text{dis}}(\mathbf{q},\omega)$. Later, we will show how the disconnected contribution can be exactly canceled out by considering only the connected scattering mechanism.

To begin, substituting $S_i^z(t) = e^{iHt} S_i^z e^{-iHt}$ in terms of the fermion operators, $\mathcal{S}_{\rm tot}(\mathbf{q},\omega)$ is easily reformulated to

$$\mathcal{S}_{\rm tot}(\mathbf{q},\omega) = \int_{-\infty}^{+\infty} \frac{dt}{8\pi N^2} e^{i\omega t} \sum_{i,j} e^{i\mathbf{q}\cdot\mathbf{r}_{ij}} \tau^z_{\sigma\sigma}\tau^z_{\sigma'\sigma'} \sum_{\sigma,\sigma'} \left\langle e^{iHt} f^\dagger_{i,\sigma} f_{i,\sigma} e^{-iHt} f^\dagger_{j,\sigma'} f_{j,\sigma'} \right\rangle . \tag{S35}$$

Before proceeding further, however, let us examine the generic structure of the quadratic spinon Hamiltonian. To do so, we consider the Bogoliubov–de Gennes (BdG) basis:

$$\psi = \Big(\psi_\uparrow, \psi_\downarrow, (\psi^\dagger_\uparrow)^{\rm T}, (\psi^\dagger_\downarrow)^{\rm T}\Big)^{\rm T};\ \psi_\sigma = \Big(f_{1,\sigma}, f_{2,\sigma}, \cdots, f_{N,\sigma}\Big). \tag{S36}$$

Any general mean-field Hamiltonian can always be expressed in the form $H = \psi^\dagger H_{\rm BdG}\psi$. The eigenvalues come in positive and negative pairs ($\pm\epsilon_{\mu,\sigma}$) and, in the diagonal basis (obtained via a $4N \times 4N$ unitary transformation U),

$$\phi = \Big(\phi_\uparrow, \phi_\downarrow, (\phi^\dagger_\uparrow)^{\rm T}, (\phi^\dagger_\downarrow)^{\rm T}\Big)^{\rm T},\ \phi_\sigma = \Big(c_{1,\sigma}, c_{2,\sigma}, \cdots, c_{N,\sigma}\Big), \tag{S37}$$

the BdG Hamiltonian takes the form

$$H^d_{\rm BdG} = U^\dagger H_{\rm BdG} U = \sum_{\mu=1}^{N}\sum_{\sigma=\uparrow,\downarrow} \epsilon_{\mu,\sigma}\left(c^\dagger_{\mu,\sigma}c_{\mu,\sigma} - c_{\mu,\sigma}c^\dagger_{\mu,\sigma}\right) = \sum_{\mu=1}^{N}\sum_{\sigma=\uparrow,\downarrow} \epsilon_{\mu,\sigma}\left(2c^\dagger_{\mu,\sigma}c_{\mu,\sigma} - 1\right). \tag{S38}$$

This construction permits us to straightforwardly interpret the Bogoliubov quasiparticles as excitations with positive energy. Thus, the Fermi level lies at zero energy, and the ground state has no Bogoliubov excitations, by definition. The associated energy eigenvalues can also be listed as

$$\Big(\cdots, \epsilon_{m,\uparrow}, \cdots, \epsilon_{m,\downarrow}, \cdots, \epsilon_{m,\uparrow}, \cdots, \epsilon_{m,\downarrow}, \cdots\Big) \equiv \Big(\cdots, \epsilon_m, \cdots, \epsilon_{m+N}, \cdots, \epsilon_{m+2N}, \cdots, \epsilon_{m+3N}, \cdots\Big), \tag{S39}$$

and this is the notation we use hereafter. In terms of the new c-fermions, the original f-fermion operators are given by

$$\begin{aligned}
f_{i,\uparrow} &= \sum_{\mu=1}^{N}\Big(U_{i,\mu}c_{\mu,\uparrow} + U_{i,\mu+N}c_{\mu,\downarrow} + U_{i,\mu+2N}c^\dagger_{\mu,\uparrow} + U_{i,\mu+3N}c^\dagger_{\mu,\downarrow}\Big),\\
f_{i,\downarrow} &= \sum_{\mu=1}^{N}\Big(U_{i+N,\mu}c_{\mu,\uparrow} + U_{i+N,\mu+N}c_{\mu,\downarrow} + U_{i+N,\mu+2N}c^\dagger_{\mu,\uparrow} + U_{i+N,\mu+3N}c^\dagger_{\mu,\downarrow}\Big),\\
f^\dagger_{i,\uparrow} &= \sum_{\mu=1}^{N}\Big(U_{i+2N,\mu}c_{\mu,\uparrow} + U_{i+2N,\mu+N}c_{\mu,\downarrow} + U_{i+2N,\mu+2N}c^\dagger_{\mu,\uparrow} + U_{i+2N,\mu+3N}c^\dagger_{\mu,\downarrow}\Big),\\
f^\dagger_{i,\downarrow} &= \sum_{\mu=1}^{N}\Big(U_{i+3N,\mu}c_{\mu,\uparrow} + U_{i+3N,\mu+N}c_{\mu,\downarrow} + U_{i+3N,\mu+2N}c^\dagger_{\mu,\uparrow} + U_{i+3N,\mu+3N}c^\dagger_{\mu,\downarrow}\Big).
\end{aligned} \tag{S40}$$

This correspondence, as we now demonstrate, enables us to build up the spin structure factors piece-by-piece.

Consider first the term $\mathcal{T}_{\sigma\sigma'} \equiv \left\langle e^{iHt} f^\dagger_{i,\sigma} f_{i,\sigma} e^{-iHt} f^\dagger_{j,\sigma'} f_{j,\sigma'}\right\rangle$ with $\sigma = \sigma' =\uparrow$. Using Eq. (S40), one can write

$$\begin{aligned}
\mathcal{T}_{\uparrow\uparrow} = \sum_{\mu,\nu,\rho,\lambda=1}^{N} \Big\langle 0 \Big| e^{iHt} &\Big(U^\star_{i,\rho}c^\dagger_{\rho,\uparrow} + U^\star_{i,\rho+N}c^\dagger_{\rho,\downarrow} + U^\star_{i,\rho+2N}c_{\rho,\uparrow} + U^\star_{i,\rho+3N}c_{\rho,\downarrow}\Big)\\
&\times \Big(U_{i,\lambda}c_{\lambda,\uparrow} + U_{i,\lambda+N}c_{\lambda,\downarrow} + U_{i,\lambda+2N}c^\dagger_{\lambda,\uparrow} + U_{i,\lambda+3N}c^\dagger_{\lambda,\downarrow}\Big) e^{-iHt}\\
&\times \Big(U^\star_{j,\mu}c^\dagger_{\mu,\uparrow} + U^\star_{j,\mu+N}c^\dagger_{\mu,\downarrow} + U^\star_{j,\mu+2N}c_{\mu,\uparrow} + U^\star_{j,\mu+3N}c_{\mu,\downarrow}\Big)\\
&\times \Big(U_{j,\nu}c_{\nu,\uparrow} + U_{j,\nu+N}c_{\nu,\downarrow} + U_{j,\nu+2N}c^\dagger_{\nu,\uparrow} + U_{j,\nu+3N}c^\dagger_{\nu,\downarrow}\Big)\Big| 0\Big\rangle.
\end{aligned} \tag{S41}$$

Recalling that the ground state is the Bogoliubov vacuum $|0\rangle$, a scattering process follows the creation of a pair of Bogoliubov quasiparticles at time 0 and their annihilation at a later time t. Therefore, the terms of the form

"$c_{\rho,\sigma_1}(t)c_{\lambda,\sigma_2}(t)c^{\dagger}_{\mu,\sigma_1'}(0)c^{\dagger}_{\nu,\sigma_2'}(0)$" will contribute to the structure factors. On the other hand, this ensures the exclusion of the disconnected contributions, i.e., $\mathcal{S}_{\rm dis}(\mathbf{q},\omega)$ in Eq. (S34), arising from terms like "$c_{\rho,\sigma_1}(t)c^{\dagger}_{\rho,\sigma_1}(t)c_{\mu,\sigma_1'}(0)c^{\dagger}_{\mu,\sigma_1'}(0)$" where the process follows the creation of quasiparticles (μ,σ_1') and (ρ,σ_1) at times 0 and t, respectively, followed by their annihilation at the same time.

The connected pieces, which we concentrate on, can be further simplified. To illustrate this, let us take, for example, the term $\langle 0|e^{iHt}c_{\rho,\uparrow}c_{\lambda,\uparrow}e^{-iHt}c^{\dagger}_{\mu,\uparrow}c^{\dagger}_{\nu,\uparrow}|0\rangle$, which contributes to the DSF as

$$\begin{aligned}
&\int_{-\infty}^{+\infty}\frac{dt}{8\pi N^2}e^{i\omega t}\sum_{i,j}e^{i\mathbf{q}\cdot\mathbf{r}_{ij}}\sum_{\mu,\nu,\rho,\lambda}U^{\star}_{i,\rho+2N}U_{i,\lambda}U^{\star}_{j,\mu}U_{j,\nu+2N}\langle 0|e^{iHt}c_{\rho,\uparrow}c_{\lambda,\uparrow}e^{-iHt}c^{\dagger}_{\mu,\uparrow}c^{\dagger}_{\nu,\uparrow}|0\rangle\\
&=\int_{-\infty}^{+\infty}\frac{dt}{8\pi N^2}e^{i\omega t}\sum_{i,j}e^{i\mathbf{q}\cdot\mathbf{r}_{ij}}\sum_{\mu,\nu,\rho,\lambda}U^{\star}_{i,\rho+2N}U_{i,\lambda}U^{\star}_{j,\mu}U_{j,\nu+2N}\langle 0|c_{\rho,\uparrow}c_{\lambda,\uparrow}c^{\dagger}_{\mu,\uparrow}c^{\dagger}_{\nu,\uparrow}|0\rangle e^{-i(\epsilon_\mu+\epsilon_{\nu+2N})t}\\
&=\frac{1}{4N^2}\sum_{i,j}e^{i\mathbf{q}\cdot\mathbf{r}_{ij}}\sum_{\mu,\nu,\rho,\lambda}U^{\star}_{i,\rho+2N}U_{i,\lambda}U^{\star}_{j,\mu}U_{j,\nu+2N}\langle 0|c_{\rho,\uparrow}c_{\lambda,\uparrow}c^{\dagger}_{\mu,\uparrow}c^{\dagger}_{\nu,\uparrow}|0\rangle\delta(\epsilon_\mu+\epsilon_{\nu+2N}-\omega)\\
&=\frac{1}{4N^2}\sum_{i,j}e^{i\mathbf{q}\cdot\mathbf{r}_{ij}}\sum_{\mu,\nu,\rho,\lambda}U^{\star}_{i,\rho+2N}U_{i,\lambda}U^{\star}_{j,\mu}U_{j,\nu+2N}(\delta_{\rho,\nu}\delta_{\lambda,\mu}-\delta_{\rho,\mu}\delta_{\lambda,\nu})\delta(\epsilon_\mu+\epsilon_{\nu+2N}-\omega)\\
&=\frac{1}{4N^2}\sum_{i,j,\mu,\nu}e^{i\mathbf{q}\cdot\mathbf{r}_{ij}}(U^{\star}_{i,\nu+2N}U_{i,\mu}-U^{\star}_{i,\mu+2N}U_{i,\nu})U^{\star}_{j,\mu}U_{j,\nu+2N}\delta(\epsilon_\mu+\epsilon_{\nu+2N}-\omega).
\end{aligned}\tag{S42}$$

One can analogously derive all the other terms constituting $\mathcal{T}_{\uparrow\uparrow}$. Combining these, the net contribution of $\mathcal{T}_{\uparrow\uparrow}$ to $\mathcal{S}(\mathbf{q},\omega)$ takes the following form:

$$\begin{aligned}
(\mathcal{S}(\mathbf{q},\omega))_{\uparrow\uparrow}=\frac{1}{4N^2}\sum_{i,j}e^{i\mathbf{q}\cdot\mathbf{r}_{ij}}\sum_{\mu,\nu}\quad&\big[(U^{\star}_{i,\nu+2N}U_{i,\mu}-U^{\star}_{i,\mu+2N}U_{i,\nu})U^{\star}_{j,\mu}U_{j,\nu+2N}\delta(\epsilon_\mu+\epsilon_{\nu+2N}-\omega)\\
&+(U^{\star}_{i,\nu+3N}U_{i,\mu+N}-U^{\star}_{i,\mu+3N}U_{i,\nu+N})U^{\star}_{j,\mu+N}U_{j,\nu+3N}\delta(\epsilon_{\mu+N}+\epsilon_{\nu+3N}-\omega)\\
&+(U^{\star}_{i,\nu+2N}U_{i,\mu+N}-U^{\star}_{i,\mu+3N}U_{i,\nu})U^{\star}_{j,\mu+N}U_{j,\nu+2N}\delta(\epsilon_{\mu+N}+\epsilon_{\nu+2N}-\omega)\\
&+(U^{\star}_{i,\nu+3N}U_{i,\mu}-U^{\star}_{i,\mu+2N}U_{i,\nu+N})U^{\star}_{j,\mu}U_{j,\nu+3N}\delta(\epsilon_{\mu}+\epsilon_{\nu+3N}-\omega)\big]\\
\equiv\frac{1}{4N^2}\sum_{i,j}e^{i\mathbf{q}\cdot\mathbf{r}_{ij}}g(i,j).&
\end{aligned}\tag{S43}$$

Carrying out a similar analysis for the other components, i.e., $(\mathcal{S}(\mathbf{q},\omega))_{\downarrow\downarrow}$, $(\mathcal{S}(\mathbf{q},\omega))_{\uparrow\downarrow}$, and $(\mathcal{S}(\mathbf{q},\omega))_{\downarrow\uparrow}$, one finds the full expression for the DSF to be

$$\mathcal{S}(\mathbf{q},\omega)=\frac{1}{4N^2}\sum_{i,j}e^{i\mathbf{q}\cdot\mathbf{r}_{ij}}[g(i,j)+g(i+N,j+N)-g(i,j+N)-g(i+N,j)].\tag{S44}$$

The static structure factor, which is the equal-time momentum-resolved spin-spin correlation function, can then directly be calculated from this equation as $S(\mathbf{q})=\sum_\omega\mathcal{S}(\mathbf{q},\omega)$.

Following the procedure outlined above, we systematically compute the static and dynamical structure factors for all our $\mathbb{Z}_2$ QSL *Ansätze*, over a broad range of parameters, and these are presented in Supplementary Figures 1 through 6, and Supplementary Figures 7 to 12, respectively.

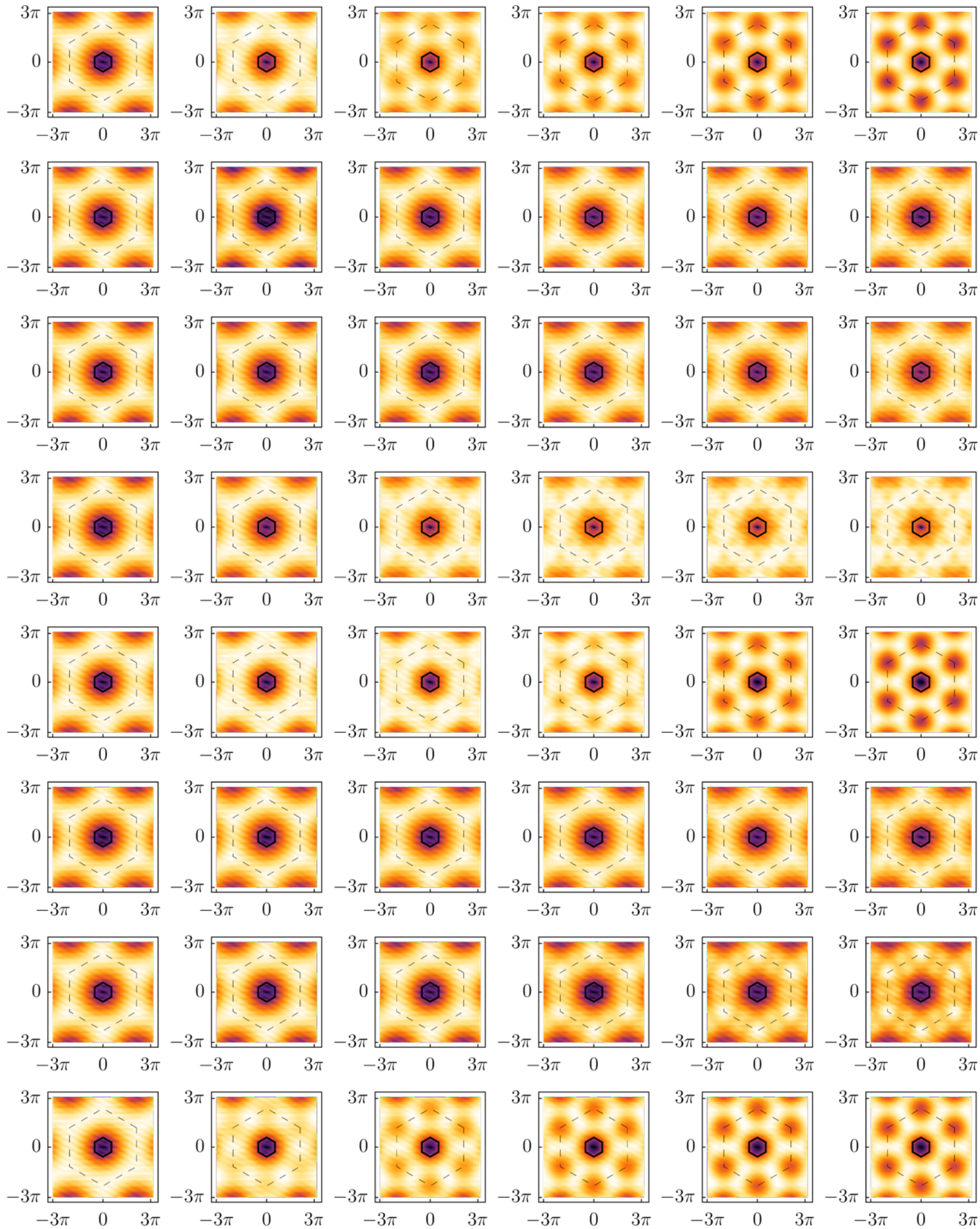

Supplementary Figure 1. Static structure factors for the $\mathbb{Z}_2$ *Ansätze* listed in the first eight rows of Table 1 of the main text, with $\eta_{C_6} = \eta = +1$, for a system size of $10 \times 10 \times 6$ sites. The mean-field parameters for the first-nearest-neighbor (1NN) and second-nearest-neighbor (2NN) bonds are chosen in the ratio $1 : r$, while those on the third-nearest-neighbor (3NN) bonds are set to zero. The six columns, from left to right, correspond to $r = 0.25$, 0.50, 0.75, 1.00, 1.25, and 1.50, respectively. Without loss of generality, we choose an onsite triplet term of magnitude 0.2. The solid (dashed) hexagon marks the first (extended) Brillouin zone.

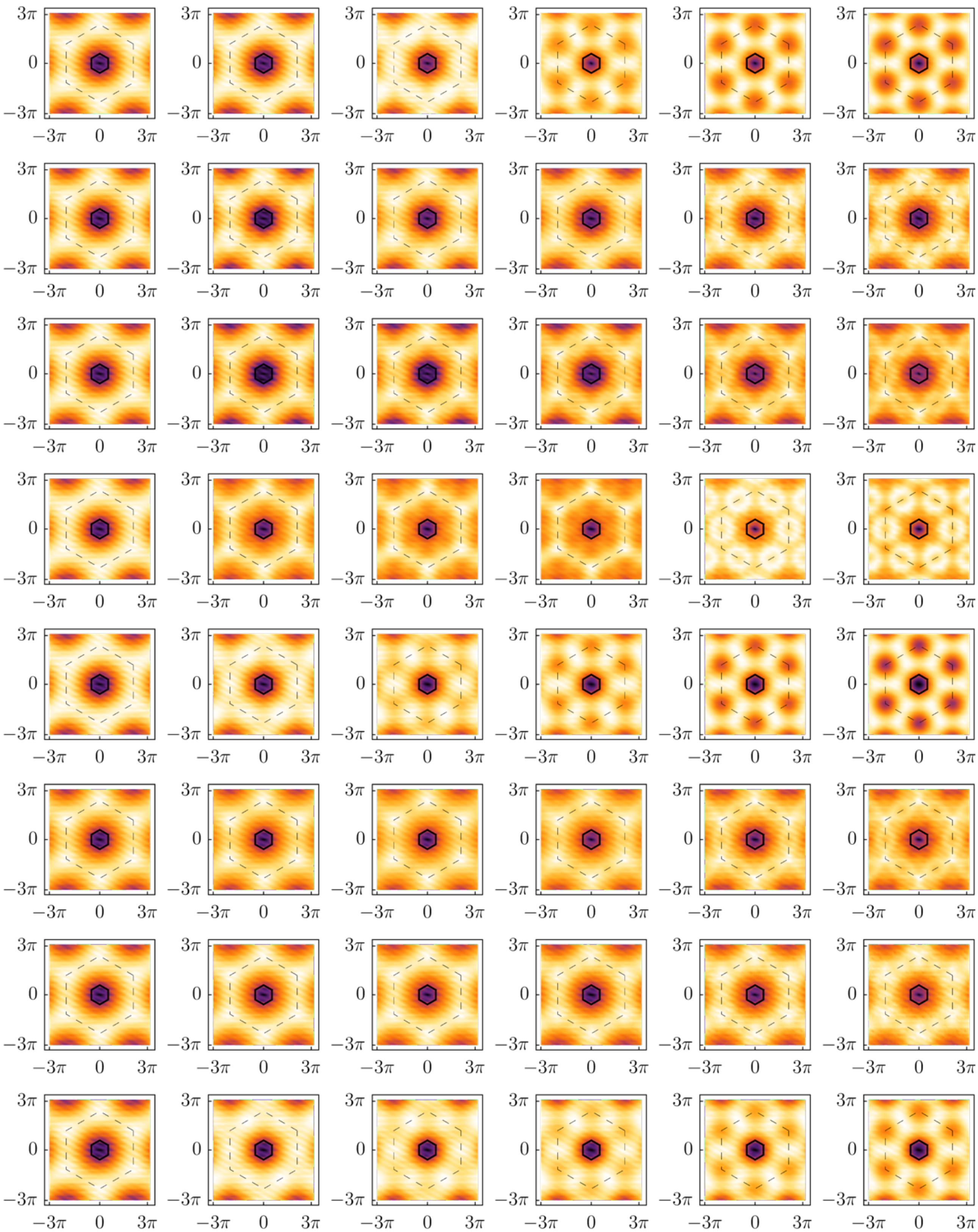

Supplementary Figure 2. Same as in Supplementary Figure 1 but for the $\mathbb{Z}_2$ *Ansätze* in the $\eta_{C_6} = \eta = -1$ class.

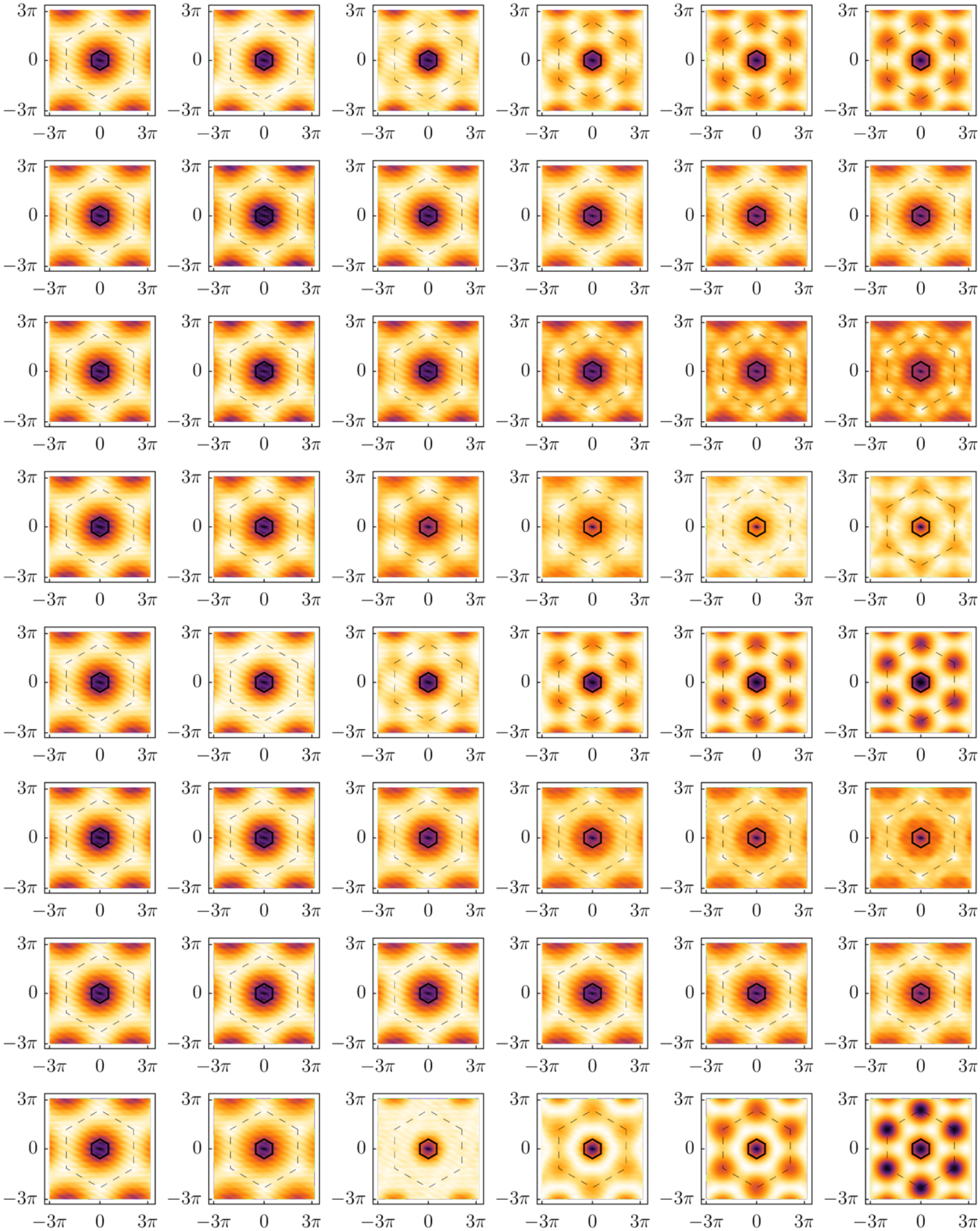

Supplementary Figure 3. Same as in Supplementary Figure 1 but for the $\mathbb{Z}_2$ *Ansätze* in the $\eta_{C_6} = -\eta = -1$ class.

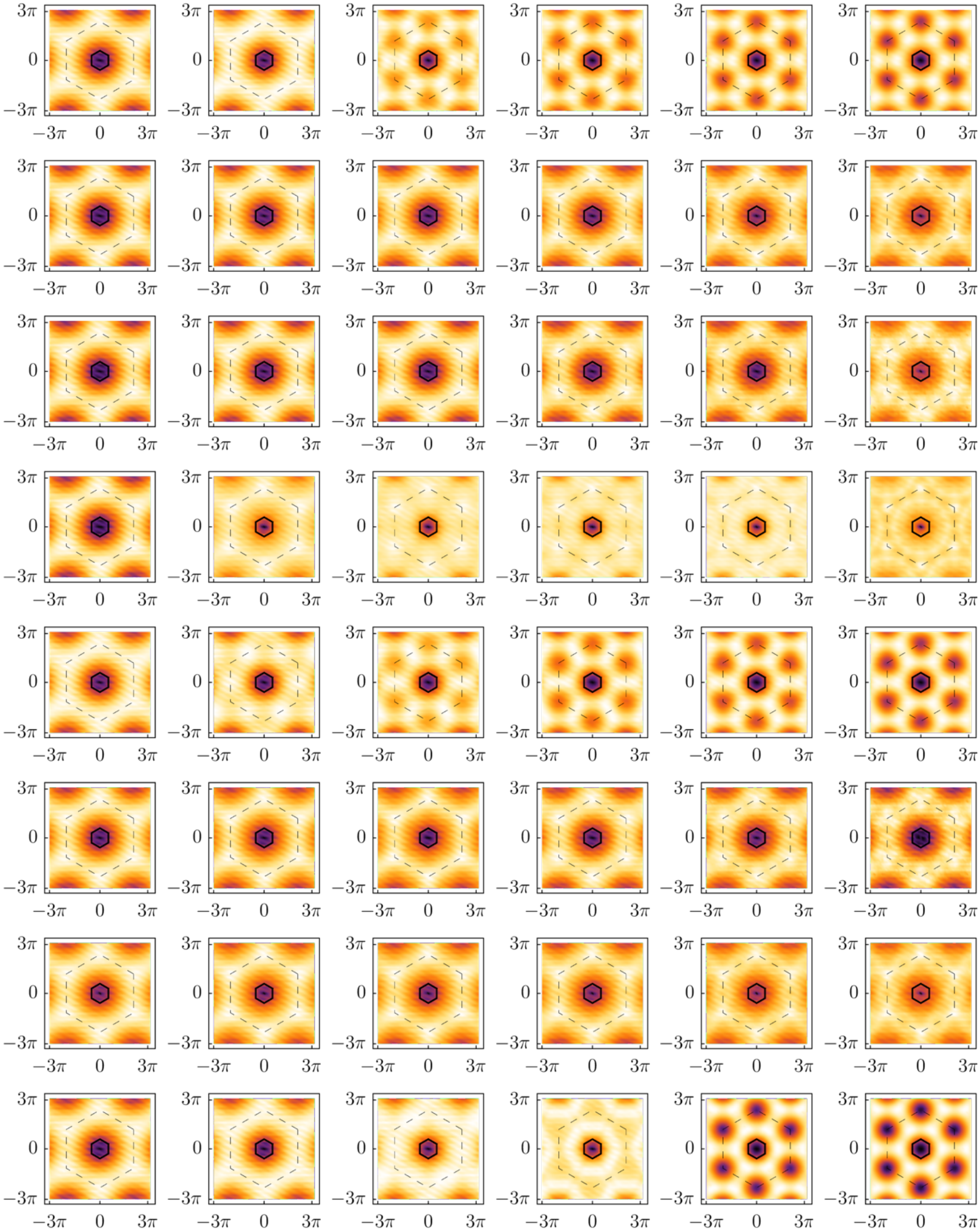

Supplementary Figure 4. Same as in Supplementary Figure 1 but for the $\mathbb{Z}_2$ *Ansätze* in the $\eta_{C_6} = -\eta = +1$ class.

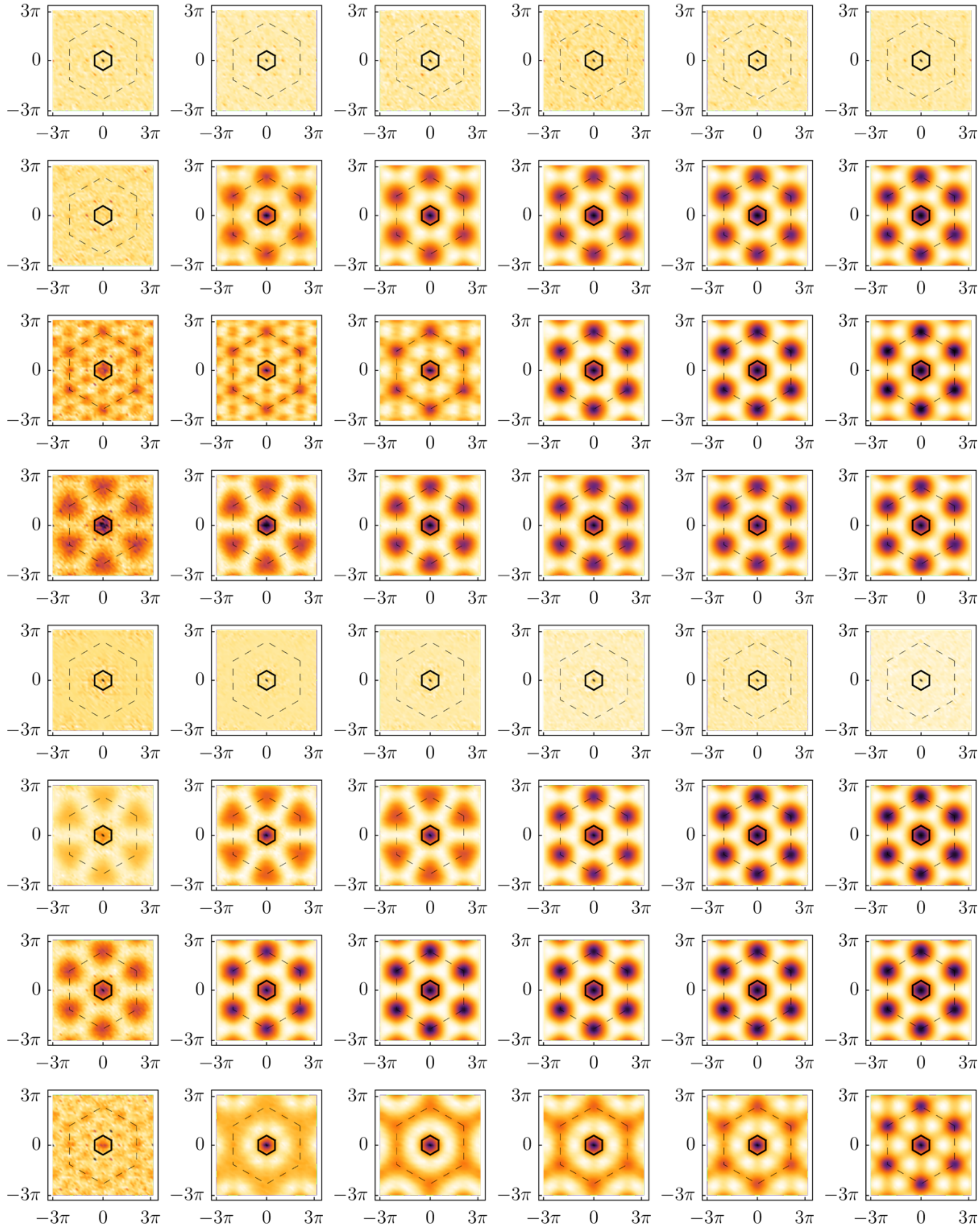

Supplementary Figure 5. Static structure factors for the $\mathbb{Z}_2$ *Ansätze* listed in rows 9–12 of Table 1 of the main text, with $\eta_{C_6} = \eta = +1$ (first four rows of the figure) and $\eta_{C_6} = -\eta = -1$ (last four rows here). All other parameters and conventions are chosen to be the same as in Supplementary Figure 1.

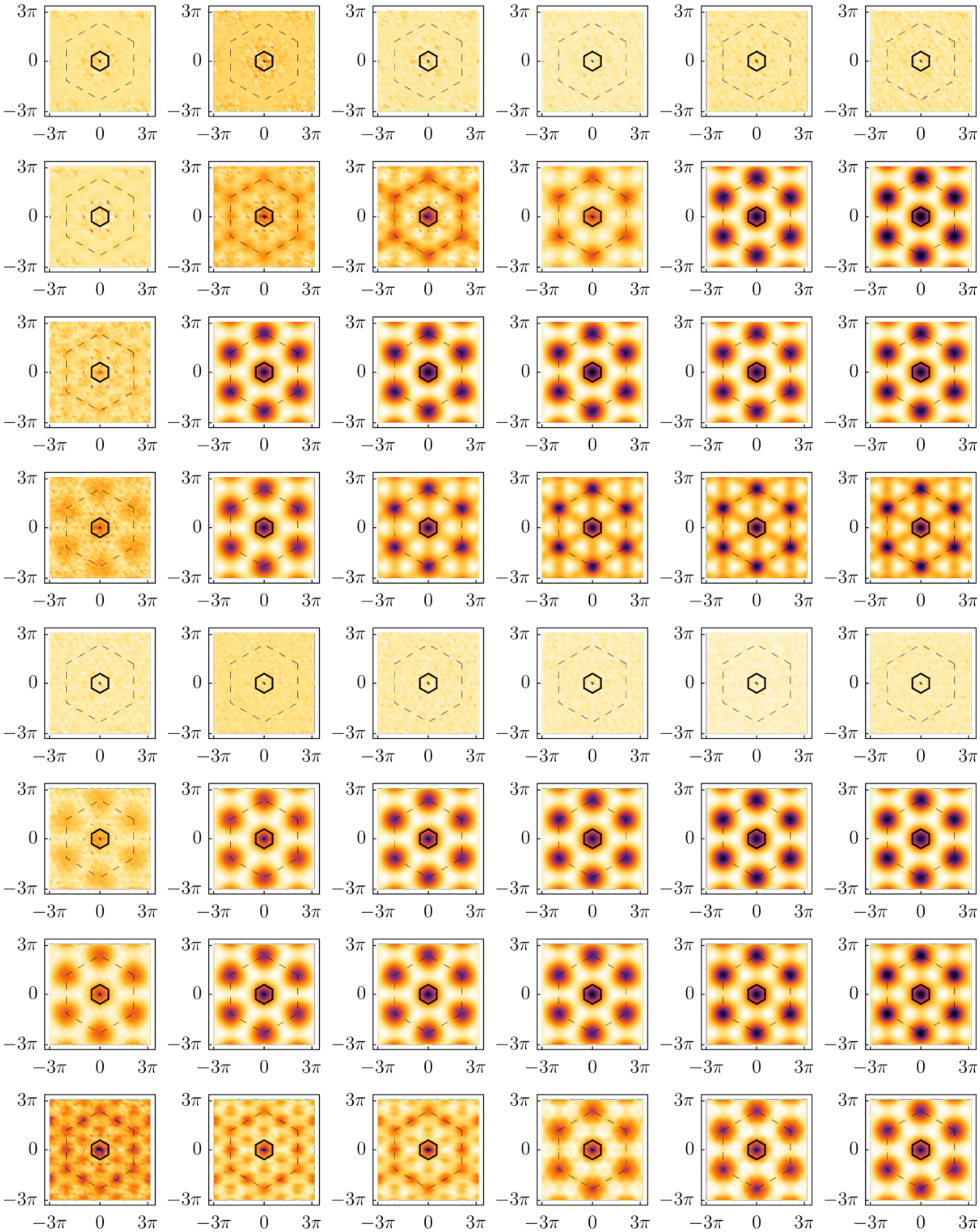

Supplementary Figure 6. Same as in Supplementary Figure 5, but for the $\eta_{C_6} = \eta = -1$ (first four rows) and $\eta_{C_6} = -\eta = +1$ (last four rows) classes.

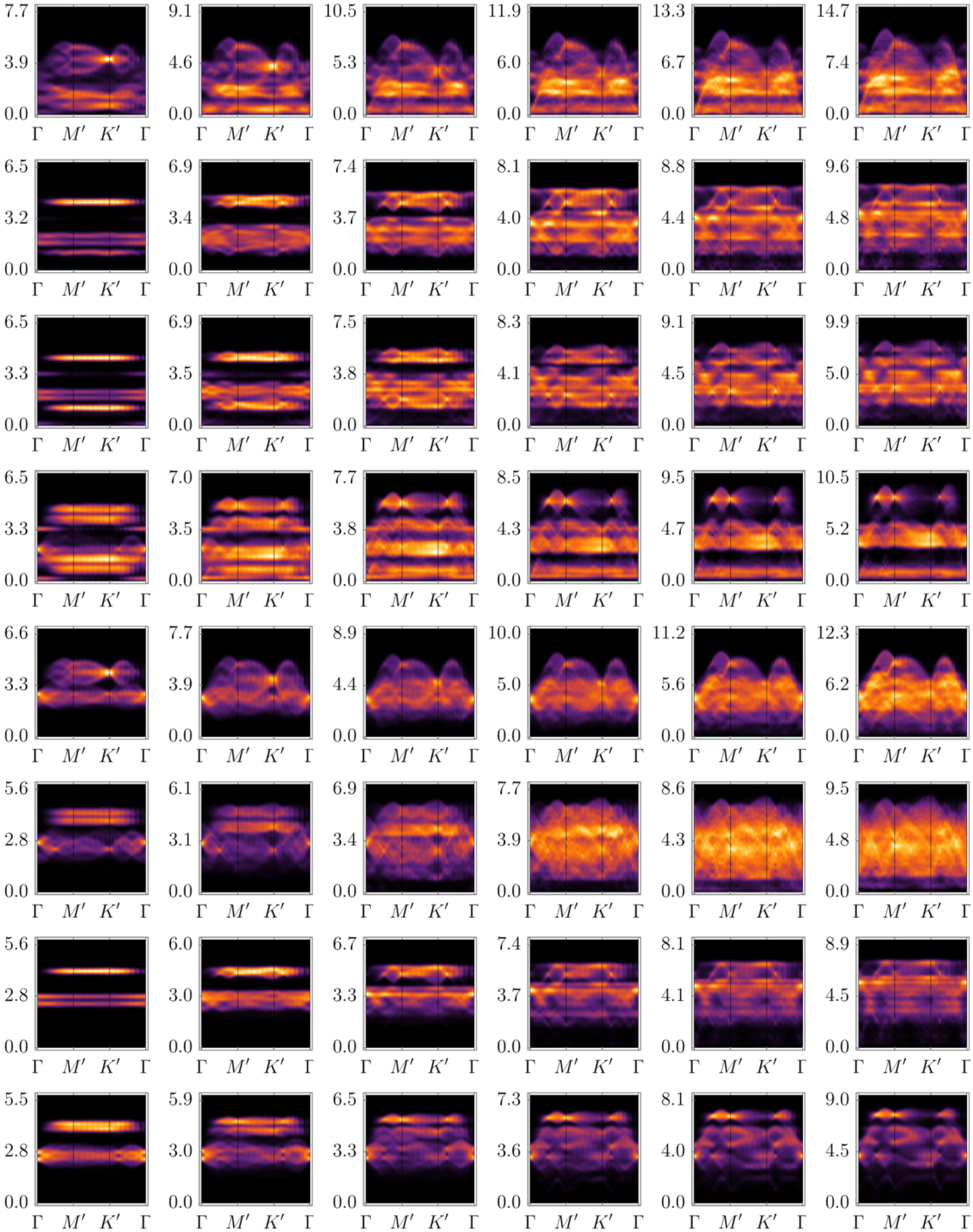

Supplementary Figure 7. Dynamical structure factors, plotted along a high-symmetry path in the extended Brillouin zone, for the $\mathbb{Z}_2$ *Ansätze* listed in the first eight rows of Table 1 of the main text, with $\eta_{C_6} = \eta = +1$. All parameters and conventions are chosen to be the same as in Supplementary Figure 1.

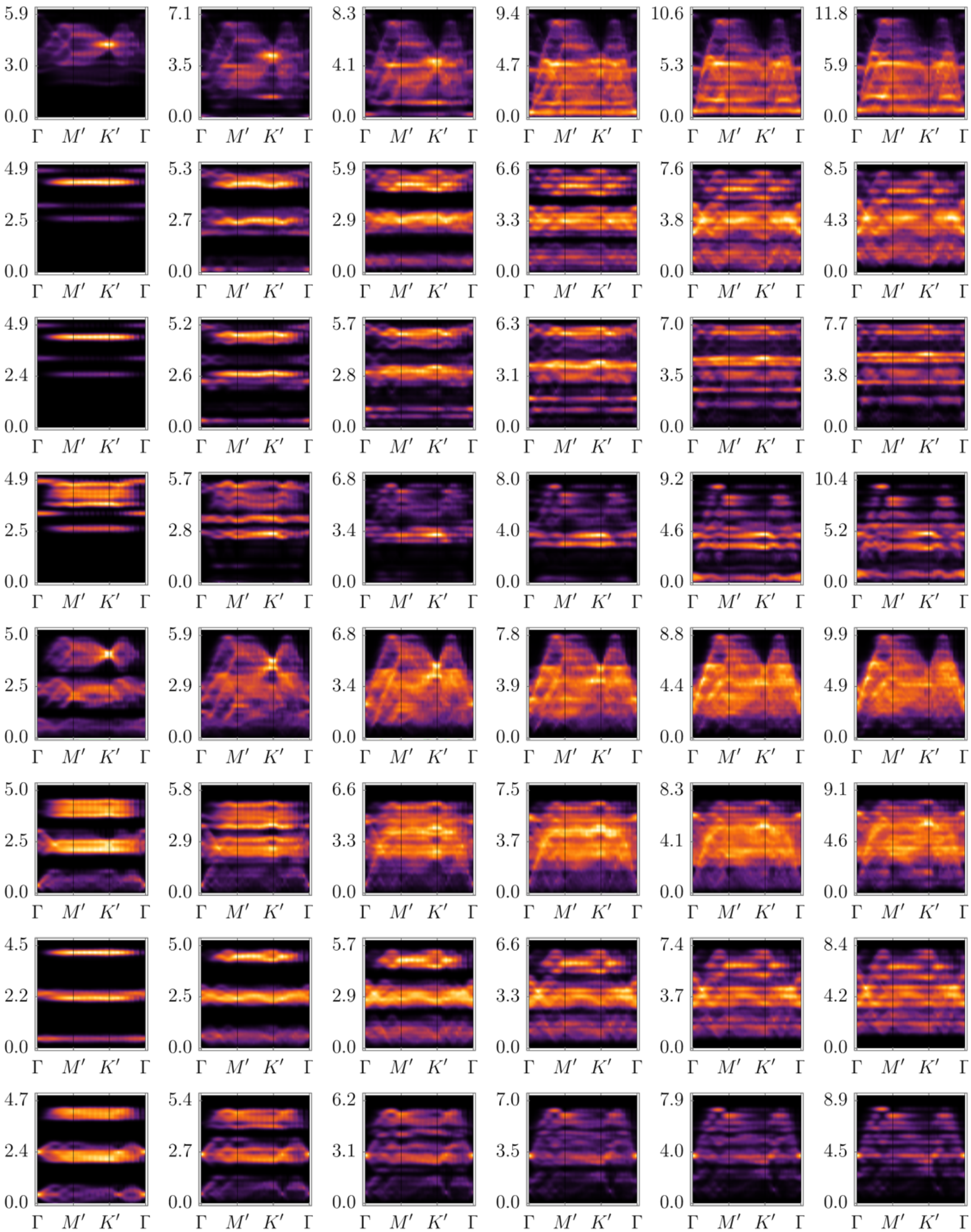

Supplementary Figure 8. Same as in Supplementary Figure 7 but for the $\mathbb{Z}_2$ *Ansätze* in the $\eta_{C_6} = \eta = -1$ class.

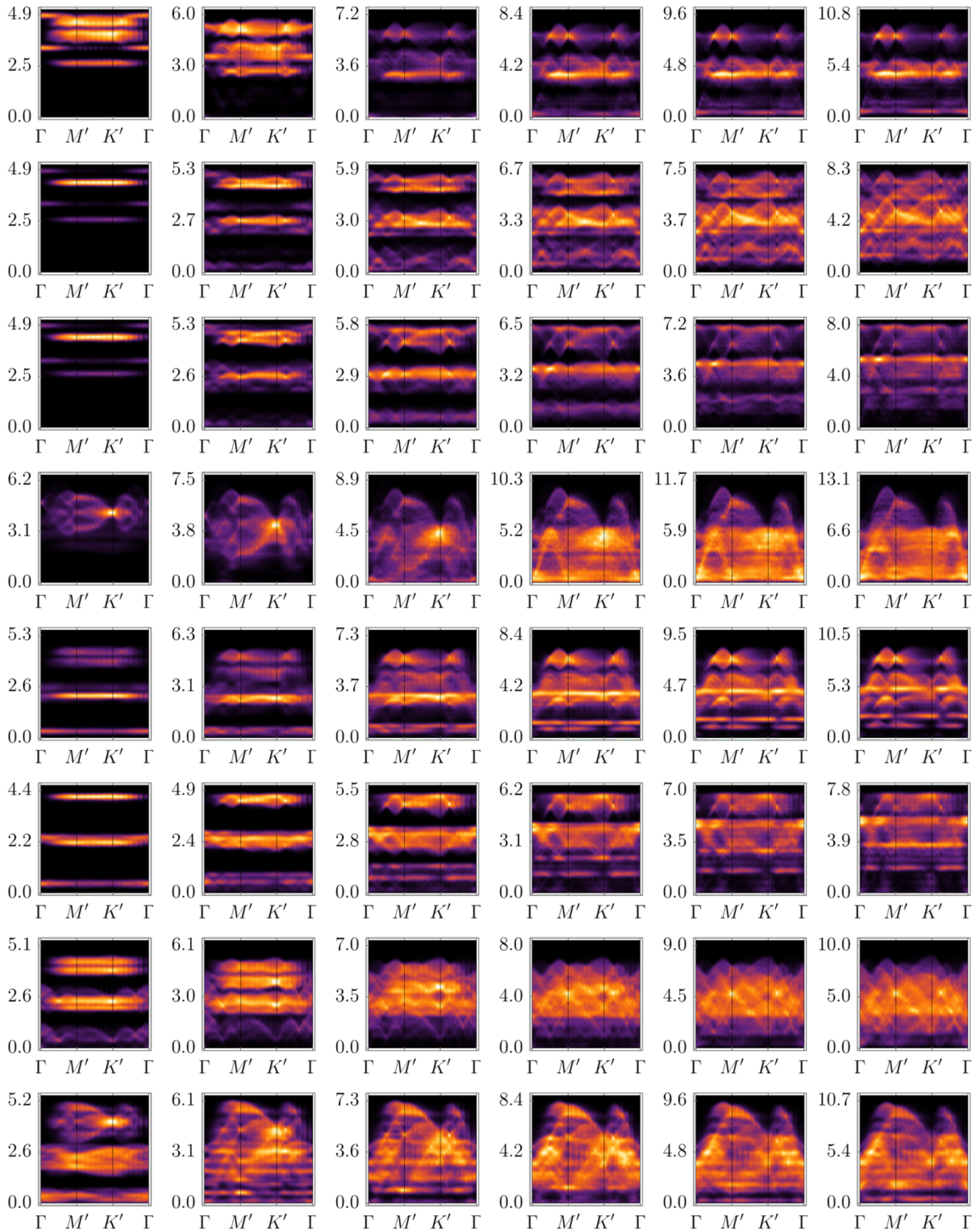

Supplementary Figure 9. Same as in Supplementary Figure 7 but for the $\mathbb{Z}_2$ *Ansätze* in the $\eta_{C_6} = -\eta = -1$ class.

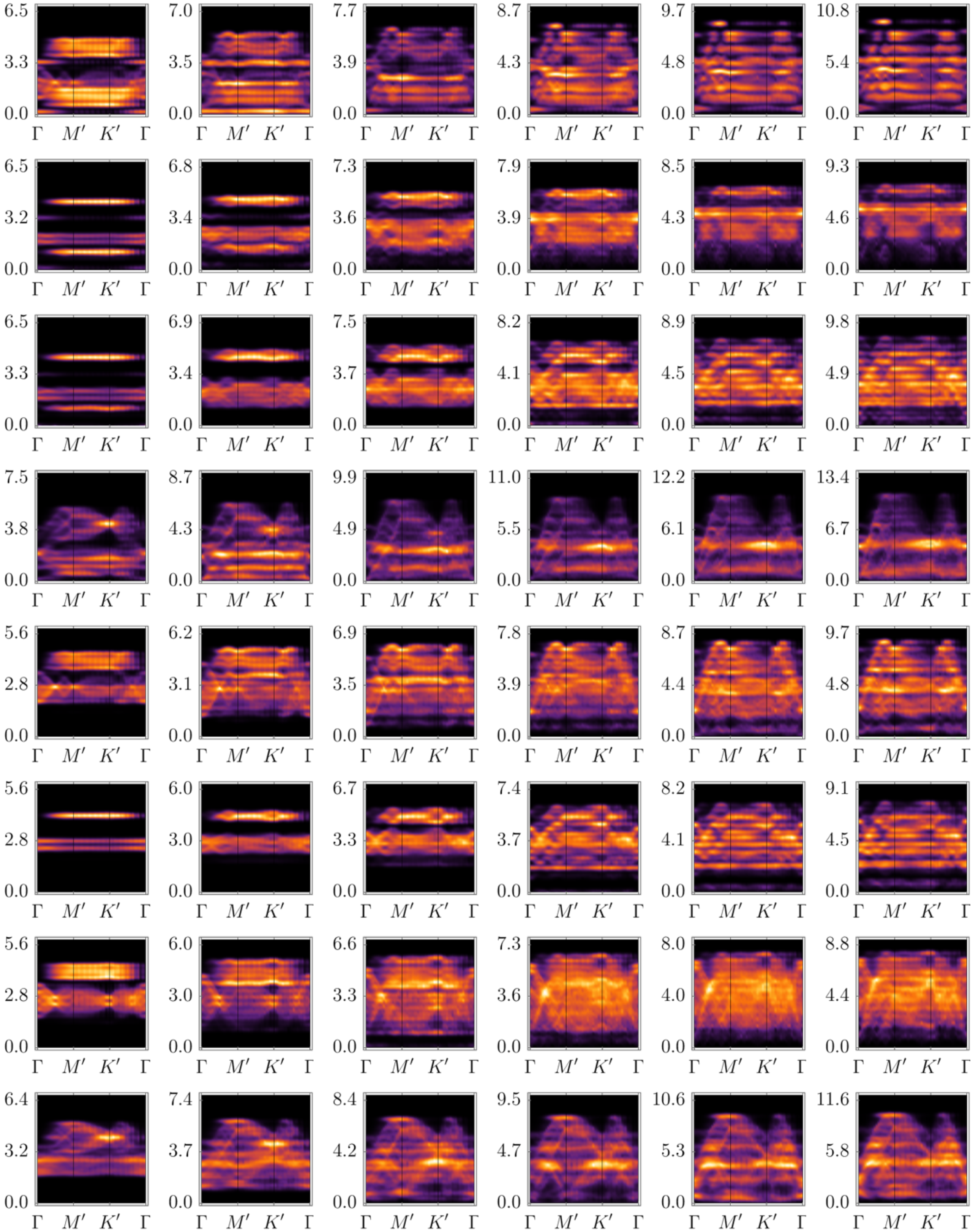

Supplementary Figure 10. Same as in Supplementary Figure 7 but for the $\mathbb{Z}_2$ *Ansätze* in the $\eta_{C_6} = -\eta = 1$ class.

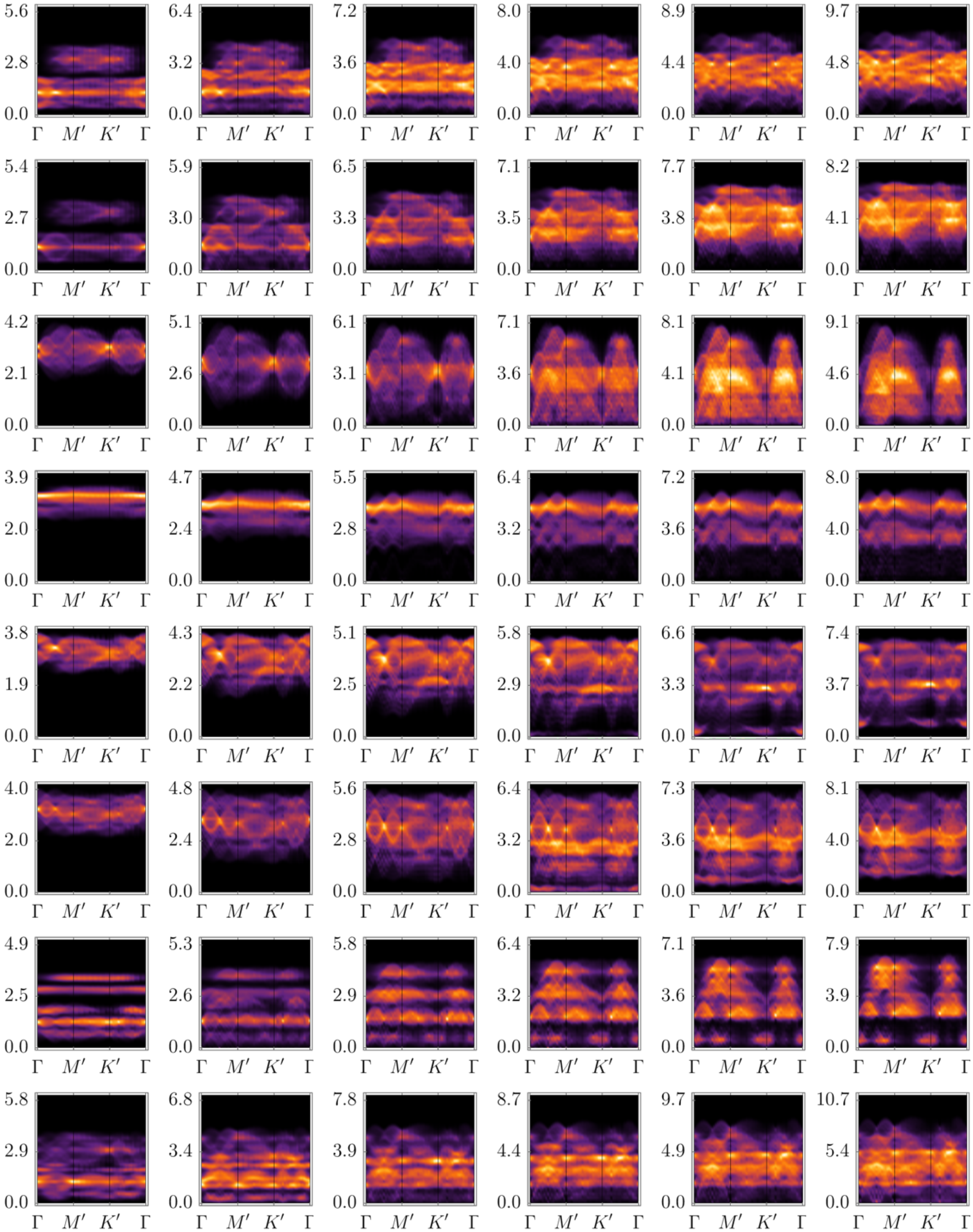

Supplementary Figure 11. Dynamical structure factors for the $\mathbb{Z}_2$ *Ansätze* listed in rows 9–12 of Table 1 of the main text, with $\eta_{C_6} = \eta = +1$ (first four rows of the figure) and $\eta_{C_6} = -\eta = -1$ (last four rows here). All other parameters and conventions are taken to be the same as in Supplementary Figure 7.

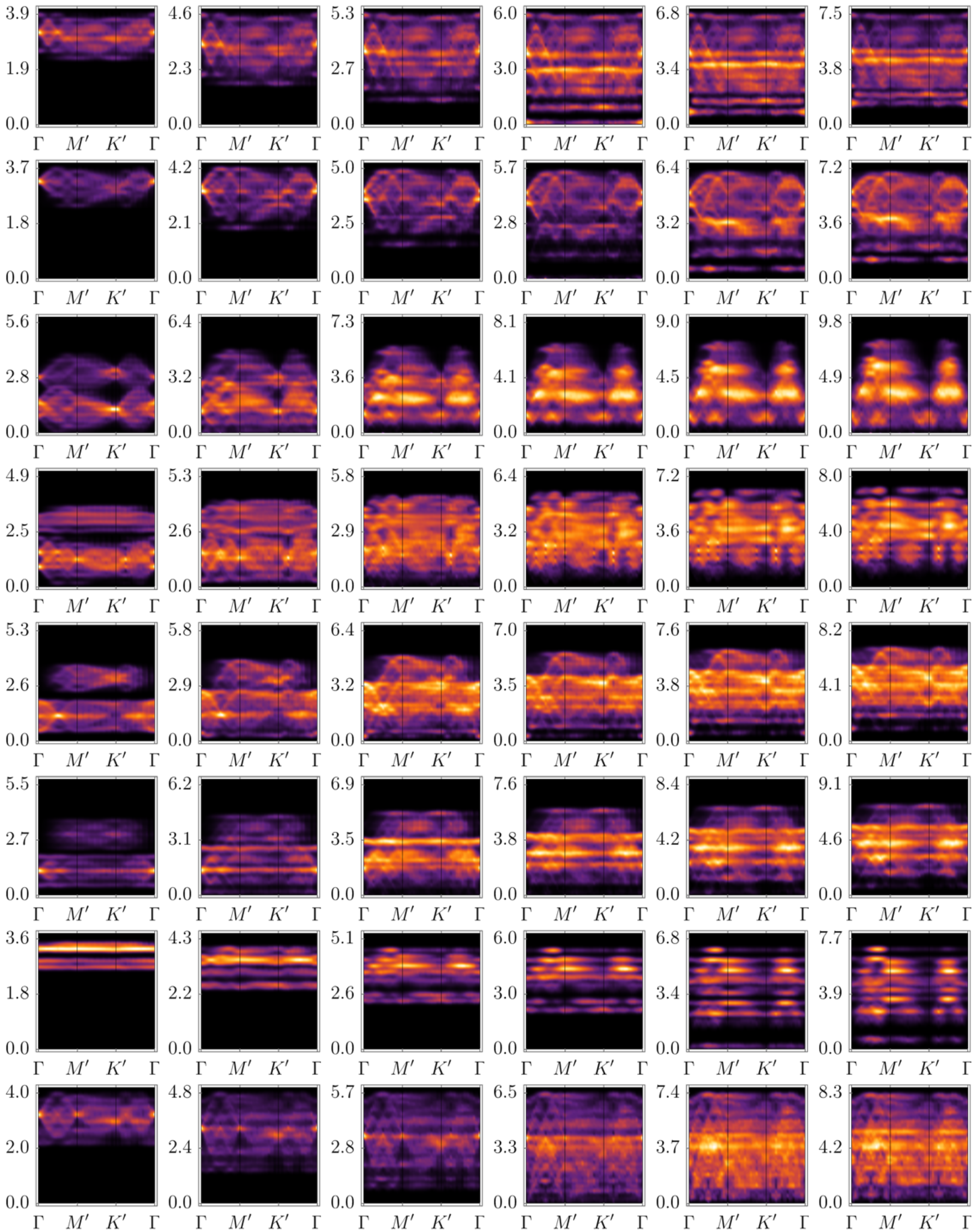

Supplementary Figure 12. Same as in Supplementary Figure 11 but for the $\eta_{C_6} = \eta = -1$ (first four rows) and $\eta_{C_6} = -\eta = +1$ (last four rows) classes.

[1] A. Maity, R. Samajdar, and Y. Iqbal, Gapped and gapless quantum spin liquids on the ruby lattice, (2024), arXiv:2409.16344 [cond-mat.str-el].
