# Supplementary material for: Fermionic parton theory of Rydberg ℤ2 quantum spin liquids
Source: Nat Commun. 2026 Jul 13;17:8588. doi: 10.1038/s41467-026-75245-9 (PMC13487239; doi:10.1038/s41467-026-75245-9)
Supplement: Supplementary file 2 [file 41467_2026_75245_MOESM2_ESM.pdf]

nature portfolio

Peer Review File

Fermionic parton theory of Rydberg $\mathbb{Z}_2$ quantum spin liquids

Corresponding Author: Professor Yasir Iqbal

This file contains all reviewer reports in order by version, followed by all author rebuttals in order by version.

Version 0:

Reviewer comments:

Reviewer #1

(Remarks to the Author)
In the manuscript "Fermionic parton theory of Rydberg Z2 quantum spin liquids", the authors are motivated by recent advances in the realization of frustrated spin models on Rydberg atom arrays. In particularly, previous experiments suggest that an effective S=1/2 model on the Ruby lattice may host a quantum spin liquid phase. The authors perform a systematic projective symmetry group classification of all possible ansatz states. They then perform a Density Matrix Renormalization Group study of the microscopic spin Hamiltonian, compute the dynamical and static spin-structure factors and compare them with results obtained from various spinon ansatz states. They are able to identify a particular Z2 spin-liquid state which matches their DMRG calculation.

The manuscript is very well written, and I enjoyed the particularly systematic approach the authors take. The results seem valid and are of value for the study and analyses of putative QSL states in Rydberg states. In particular, the broken spin-rotation invariance in these systems requires one to revisit a PSG classification of these states. However, I have doubts whether the reported methods or results are novel enough, or of sufficiently big impact, to warrant a publication in Nature Communications. I suggest that the authors make more explicit why they feel their work constitutes a significant advancement?

I have a few more detailed questions:

1) Could the authors elaborate on the relation between the work at hand and their reference 35, where a PSG classification of QSL states on the Ruby lattice seems to have been undertaken already? Are the results in Table I a reproduction of this work?

2) The authors claim that Ref. 26 established that the Ruby lattice hosts a QSL state experimentally. My understanding that this is still a point of controversy and there is no clear consensus on this, or whether the observed behaviour may be a non-equilibrium feature. Similar issues arise in dipolar XY models simulated by Rydberg atom arrays [see, for example, Sbierski et al., PRB 109, 144411 (2024)]. Could the authors expand on this point?

3) In the introduction, the authors establish the fundamental notion of Z2 topological order in terms of four distinct quasiparticle sectors (1, e, m, epsilon). However, the fermionic spinon theory appears to only consider fermionic excitations. In the conclusion, the authors allude to a Schwinger-boson classification, probably to access the e-particle sector? I believe that in recent years, there has been some progress (at least in some cases) in understanding how PSG classifications are tied to a classification of topological order, see e.g. PRB 96, 205150 (2017). I believe it would be helpful for the authors to put their methodology into this context.

4) What parameter values of \Omega, \Delta, V_ij have been used for the DMRG simulations in Fig. 2(a)? How do these parameters relate to the experiment of Ref. 26?

5) In a similar vein, with a DMRG study of the Rydberg Ruby lattice Hamiltonian at hand, have the authors verified that the ground state indeed hosts Z2 topological order? (This should be fairly straightforward, by measuring the entanglement entropy of the state).

6) On a theoretical level, I wonder if comparing structure factors is really a useful quantity to identify the QSL. These are notoriously hard to measure in Rydberg atom arrays. Are there any other, more direct observables? Could one use projective measurements of some string order parameters as in Ref. 26, or some quench spectroscopy method (that provides access to the one-time correlation function)?

(Remarks on code availability)

Reviewer #2

(Remarks to the Author)
Dear Editor,

In this manuscript, Maity et al. presents a systematic classification of possible Z_2 quantum spin liquid (QSL) states on the ruby lattice using fermionic parton mean-field theory and projective symmetry group (PSG) analysis. The authors further compute static and dynamical structure factors for all candidate ansatze, and identify a promising state consistent with their DMRG results.

In my opinion, this is a important and innovative work. The manuscript is well-structured and and offers a valuable theory for understanding the QSLs in Rydberg atom arrays. I believe it is suitable for publication in Nature Communications after addressing the points outlined below.

Major:

1.The role of gauge fluctuations and their negligible effect on observable quantities could be explained more intuitively for a broader readership.
2.The comparison of SSF/DSF is good. Maybe the author can discuss whether other observables (e.g., entanglement entropy, topological excitation spectra) could further distinguish between ansatz.
3.It would be good if briefly comment on whether Gutzwiller projection could qualitatively alter the structure factor predictions.
4.It would be helpful if provide typical experimental parameter ranges (Ω, Δ, V_0) in the main text would enhance practical relevance.

Minor:
1.The authors should provide details data of DMRG simulations, e.g., the lattice/geometry used in DMRG, the bond dimension, the converged energy, etc.

(Remarks on code availability)

Reviewer #3

(Remarks to the Author)
In this paper, the authors employ the projective symmetry group (PSG) framework to characterize symmetry fractionalization patterns in a topologically ordered Z2 quantum spin liquid (QSL) realized in a Rydberg atom array on the ruby lattice. They identify 64 Z2 projective extensions of the symmetry group, among which 50 are claimed to be realizable with a Z2 invariant gauge group (IGG). The authors further argue that these 50 possibilities are experimentally relevant and outline a concrete route for realizing them in the laboratory.

This analysis is certainly interesting and physically well motivated. Personally, what I find most compelling are the static and dynamical structure factors presented in the paper, which provide clear signatures that can be probed both numerically and experimentally. I think it is overall a nice paper with significant scientific importance, and I would like to recommend its publication should the authors address my comments properly.

The authors state that the PSG calculation must be carried out up to third-nearest-neighbor (3NN) bonds. Is this strictly necessary? What changes if one truncates the analysis at second-nearest neighbors (2NN), or alternatively extends it to fourth-nearest neighbors (4NN)? I suspect that the geometry of the ruby lattice imposes a natural cutoff, but this is not entirely clear from the current presentation.

Much of the classic PSG literature focuses on systems with strictly local interactions. In the present setup, the interaction V is long-ranged. Could the authors clarify whether, and if so how, the long-range nature of V plays a role or not in the PSG classification?

I believe that the diagrams for the static and dynamical structure factors presented in the paper are calculated from the mean field ansatz. While this is a natural assumption, the strongly-coupling nature of the system may have significant impact on the results. I wonder what is the authors' perspective on how the effect beyond mean-field theory may distort these diagrams or not.

Also, I would also like to draw the authors' attention to several related works. In https://arxiv.org/pdf/1507.07621, the authors carry out a PSG classification for the same symmetry group and also obtain 64 solutions. It would be helpful if the present manuscript cited this work and commented on whether the agreement in the number of PSG solutions is coincidental or reflects a deeper structural reason. Furthermore, as emphasized in https://arxiv.org/abs/1410.4540
, identifying symmetry fractionalization solely through its action on the e particles (as is done in standard PSG constructions) is generally insufficient. Some discussion of this point in the context of the present work would strengthen the manuscript. Relatedly, the more recent work https://arxiv.org/abs/2309.15118 provides a systematic approach to diagnosing symmetry fractionalization of the m particles; it would be interesting to hear the authors' perspective on the relation between these results and PSG calculations presented in this manuscript.

(Remarks on code availability)

Version 1:

Reviewer comments:

Reviewer #1

(Remarks to the Author)
The authors have addressed my comments and questions in a satisfactory manner. In particular, they have clarified relations to their earlier works which also highlights the significance of their new results. I therefore recommend the paper for publication in Nat. Comms.

(Remarks on code availability)
I could not find any openly available code or data.

Reviewer #2

(Remarks to the Author)
Dear Editor,

I have carefully reviewed this resubmission and I appreciate the authors' professional point-by-point reply. They have addressed all the concerns raised my questions.

In this new version, the authors have stressed the distinction between this work and previous work, e.g., Ref. 35. By including the triplet channels, this work provides a needed classification for the Rydberg systems. The authors provided a robust justification for the validity of the mean-field theory in gapped Z2 QSL, which is sound. The inclusion of topological entanglement entropy data from DMRG within the reply and the listing of relevant experimental parameters strengthen the paper's quality.

The field of Rydberg-atom quantum simulation is moving fast toward identification of topological quantum phases. The manuscript provides valuable roadmap for identifying different Z2 QSLs, which bridging the gap between abstract PSG and concrete candidate states matching experimental observables. Given this, I recommend the paper for publication.

Best

(Remarks on code availability)

Reviewer #3

(Remarks to the Author)
I thank the authors for their careful reply to my comments and for the clarification of my confusing points. I think that the authors have properly addressed all of my questions, and it is my pleasure to recommend the paper's publication in Nature Communications.

(Remarks on code availability)

Open Access This Peer Review File is licensed under a Creative Commons Attribution 4.0 International License, which permits use, sharing, adaptation, distribution and reproduction in any medium or format, as long as you give appropriate credit to the original author(s) and the source, provide a link to the Creative Commons license, and indicate if changes were made.
In cases where reviewers are anonymous, credit should be given to 'Anonymous Referee' and the source.
The images or other third party material in this Peer Review File are included in the article's Creative Commons license, unless indicated otherwise in a credit line to the material. If material is not included in the article's Creative Commons license and your intended use is not permitted by statutory regulation or exceeds the permitted use, you will need to obtain permission directly from the copyright holder.
To view a copy of this license, visit https://creativecommons.org/licenses/by/4.0/

Response to Referee 1

In the manuscript "Fermionic parton theory of Rydberg $\mathbb{Z}_2$ quantum spin liquids", the authors are motivated by recent advances in the realization of frustrated spin models on Rydberg atom arrays. In particular, previous experiments suggest that an effective $S = 1/2$ model on the ruby lattice may host a quantum spin liquid phase. The authors perform a systematic projective symmetry group classification of all possible Ansatz *states. They then perform a density matrix renormalization group study of the microscopic spin Hamiltonian, compute the dynamical and static spin-structure factors and compare them with results obtained from various spinon* Ansatz *states. They are able to identify a particular $\mathbb{Z}_2$ spin-liquid state which matches their DMRG calculation.*

Response: We thank the referee for a careful reading of the paper and for a concise and accurate summary of our work.

The manuscript is very well written, and I enjoyed the particularly systematic approach the authors take. The results seem valid and are of value for the study and analyses of putative QSL states in Rydberg states. In particular, the broken spin-rotation invariance in these systems requires one to revisit a PSG classification of these states. However, I have doubts whether the reported methods or results are novel enough, or of sufficiently big impact, to warrant a publication in Nature Communications. I suggest that the authors make more explicit why they feel their work constitutes a significant advancement?
I have a few more detailed questions:

Response: We thank the referee for the positive assessment of the manuscript and for the constructive suggestion regarding the articulation of the novelty of our work. We have substantially revised the introduction to make the significant advancement clearer. As we now state, this work represents (i) the first complete PSG classification of $\mathbb{Z}_2$ QSLs on the ruby lattice in the absence of SU(2) spin-rotation symmetry—which is qualitatively richer than the SU(2)-symmetric case of Ref. [35] and necessary to describe the Rydberg platform; (ii) the identification of a specific candidate state for the microscopic Rydberg $\mathbb{Z}_2$ QSL via a systematic matching of mean-field structure factors against large-scale DMRG calculations; and (iii) a comprehensive catalog of static and dynamical structure factors for all 50 *Ansätze*, which will serve as a reference for future Rydberg-array experiments. We address the referee's detailed questions point-by-point below.

1. *Could the authors elaborate on the relation between the work at hand and their reference 35, where a PSG classification of QSL states on the ruby lattice seems to have been undertaken already? Are the results in Table I a reproduction of this work?*

   Response: We thank the referee for raising this important point, which gives us the opportunity to sharpen the contrast between the two works.

   In Ref. [35], the PSG classification was performed for a Heisenberg spin model on the ruby lattice, assuming the full symmetry group given by the lattice space group combined with *global* SU(2) spin-rotation symmetry. Consequently, only singlet mean-field channels were considered, and the resulting PSG solutions are restricted to SU(2) spin-rotation-invariant QSL states.

   In contrast, the present work addresses a fundamentally different physical setting motivated by the QSL realized on the Rydberg-atom platform. In this case, the effective spin Hamiltonian explicitly breaks SU(2) spin-rotation symmetry. On top of this, we focus on the physically relevant regime in which $\langle S_i^x \rangle = 0$ (due to the absence of e-particle condensation in the QSL ground state); this leads to an emergent $\mathrm{U}(1)_{\mathrm{spin}}$ symmetry at the mean-field level, rather than the full SU(2) of Ref. [35].

Accordingly, our PSG classification is carried out based on the lattice space-group symmetry together with $\mathrm{U}(1)_{\mathrm{spin}}$. Because of this reduced spin symmetry, additional symmetry-allowed mean-field terms—specifically the longitudinal triplet channels u^z_{ij}—must be included, and these are absent in Ref. [35]. Moreover, the effective "spin" degrees of freedom in the Rydberg platform do not correspond to physical spins; their symmetry transformation properties under spatial operations therefore differ from those of conventional spin models, and this further distinguishes the present PSG analysis from that of Ref. [35].

As a result of these differences in symmetry structure, physical motivation, and allowed mean-field *Ansätze*, the classification presented here is not only not a reproduction of Ref. [35] but it is also significantly more complex and expansive than the SU(2)-symmetric case considered therein. Table I of the present manuscript is new and contains classes of $\mathbb{Z}_2$ QSLs that do not appear in Ref. [35]. We have made this contrast explicit in the revised manuscript.

2. *The authors claim that Ref. 26 established that the ruby lattice hosts a QSL state experimentally. My understanding is that this is still a point of controversy and there is no clear consensus on this, or whether the observed behaviour may be a non-equilibrium feature. Similar issues arise in dipolar XY models simulated by Rydberg atom arrays [see, for example, Sbierski et al., PRB 109, 144411 (2024)]. Could the authors expand on this point?*

   Response: The referee is correct that the interpretation of the experiment of Ref. [26] is subtle and remains a matter of ongoing discussion. In particular, as pointed out in Ref. [23], the Rydberg QSL can be destabilized by the long-range tails of the van der Waals interaction: if the $1/r^6$ interactions are truncated beyond a finite range, the true ground state is a QSL; if the tails are retained to arbitrarily long distances, the QSL window closes and the system instead undergoes a direct first-order transition from the paramagnet to a valence-bond solid.

   For the experiment of Ref. [26], an important additional ingredient is the finite coherent lifetime before dephasing sets in. A finite observation time Δt imposes an energy-resolution scale of order $\hbar/\Delta t$ by virtue of the Heisenberg uncertainty principle; interaction effects associated with sufficiently small energy scales therefore cannot be resolved within the experimental window. As a consequence, although the exact ground state of the idealized long-range model may not be a QSL, the experiment can nevertheless display QSL-like behavior on accessible timescales, as signaled by the finite expectation value of topological loop operators measured in Ref. [26]. We agree with the referee that, from this perspective, the observed phenomenology can be viewed, at least in part, as a nonequilibrium signature of the proximate QSL phase, analogous in spirit to the discussion in the reference pointed out by the referee.

   Importantly, this subtlety does not affect our work. We consider a model in which the $1/r^6$ interactions are truncated beyond third-nearest neighbors, for which a stable $\mathbb{Z}_2$ QSL phase is well established in the ground state. This phase is the same as the QSL phase observed in the longer-range model up to the point where it is destabilized, and is therefore the relevant target for the experimental observations—both in their equilibrium and in their finite-time, nonequilibrium aspects.

   We have clarified this point in the revised manuscript and thank the referee for highlighting the need for a more careful explanation.

3. *In the introduction, the authors establish the fundamental notion of $\mathbb{Z}_2$ topological order in terms of four distinct quasiparticle sectors (1, e, m, ε). However, the fermionic spinon theory appears to only consider fermionic excitations. In the conclusion, the authors allude to a Schwinger-boson classification, probably to access the e-particle sector? I believe that in recent years, there has*

been some progress (at least in some cases) in understanding how PSG classifications are tied to a classification of topological order, see e.g. PRB 96, 205150 (2017). I believe it would be helpful for the authors to put their methodology into this context.

Response: We thank the referee for this insightful comment and for drawing our attention to the broader context connecting PSG classifications to symmetry-enriched topological order. We agree that clarifying the scope and limitations of our approach in this regard is important, and we have now done so in the manuscript.

Our fermionic parton (spinon) construction naturally captures the *fermionic* quasiparticle sector—i.e., the ε particle—of a $\mathbb{Z}_2$ topologically ordered phase. As is standard in fermionic PSG approaches, the bosonic e and m sectors are not treated on the same footing within this framework.

We note that the unified treatment of bosonic and fermionic mean-field descriptions of Ref. PRB 96, 205150 (2017) was developed for kagome-lattice spin liquids, where all quasiparticle sectors (e, m, and ε) reside on the same lattice and are related by symmetry fractionalization in a unified manner. In contrast, for the Rydberg QSL considered here, the emergent quasiparticles live on different lattices: the e, m, and ε particles reside on the kagome, dice, and ruby lattices, respectively (please see Ref. [39] for details). As a consequence, a direct application of the unified framework of PRB 96, 205150 (2017) is not straightforward in the present setting.

Accessing the e and m sectors in the Rydberg spin liquid therefore requires independent bosonic PSG classifications on the appropriate lattices. The bosonic PSG classification on the kagome lattice has been carried out in Ref. [67], though its application to the specific effective model relevant for the e-particle crystal in the Rydberg platform has not yet been addressed. Similarly, a bosonic PSG analysis on the dice lattice would be required to describe the vison (m) sector [Refs. 68, 69]; developing this classification, and studying possible m-particle crystals in this setting, is an important direction for future study.

Within this broader perspective, our fermionic PSG classification on the ruby lattice should be viewed as a systematic characterization of the ε-particle sector appropriate to the symmetry structure and microscopic constraints of the Rydberg QSL, rather than as a complete classification of all three $\mathbb{Z}_2$ quasiparticle sectors. We have added a remark in the revised manuscript putting our methodology in this context.

4. *What parameter values of Ω, Δ, V_{ij} have been used for the DMRG simulations in Fig. 2(a)? How do these parameters relate to the experiment of Ref. 26?*

   Response: We use $\Delta/\Omega = 1.7$ and take $V_{ij}/\Omega = 50$ for all pairs up to third-nearest neighbors, with the van der Waals interaction truncated beyond this range. This choice may be viewed as a generalization of the celebrated PXP model: it enforces that sites up to third-nearest neighbors are effectively blockaded, which in turn allows the low-energy subspace of the Rydberg Hamiltonian to be mapped onto a kagome-lattice dimer model that hosts the $\mathbb{Z}_2$ QSL phase. We have added the parameter values explicitly to the revised manuscript.

   The Hamiltonian used in our DMRG calculations is the standard effective model employed in prior studies of the Rydberg $\mathbb{Z}_2$ spin liquid (see, e.g., Ref. [27]). Our aim is not to reproduce the precise microscopic parameters of the experiment of Ref. [26], but rather to characterize the *universal* properties of the QSL phase. In this sense, the parameters are chosen to place the system robustly inside the same phase as the experiment, rather than to provide a quantitative fit to it. We note the excellent agreement between our DMRG static structure factor and that reported for the related kagome-lattice dimer-model study of Hwang, *Phys. Rev. B* 110, 014426 (2024), indicating that we indeed obtain the same phase numerically.

5. *In a similar vein, with a DMRG study of the Rydberg ruby lattice Hamiltonian at hand, have the authors verified that the ground state indeed hosts $\mathbb{Z}_2$ topological order? (This should be fairly straightforward, by measuring the entanglement entropy of the state).*

   Response: We thank the referee for this suggestion. The $\mathbb{Z}_2$ topological order of the Rydberg QSL ground state has been investigated in several prior works, which is why we had not originally revisited this point in the manuscript. However, one of the present authors has computed the topological entanglement entropy for the same microscopic Hamiltonian in an earlier study [Chen *et al.*, *PNAS* 122, e2416111122 (2025)], and we reproduce the corresponding result here for the referee's convenience. As shown in Fig. 1 below, the topological entanglement entropy saturates to $\gamma \approx \ln 2$ over a broad parameter range, the hallmark of $\mathbb{Z}_2$ topological order. We have added a brief remark citing this evidence in the revised manuscript.

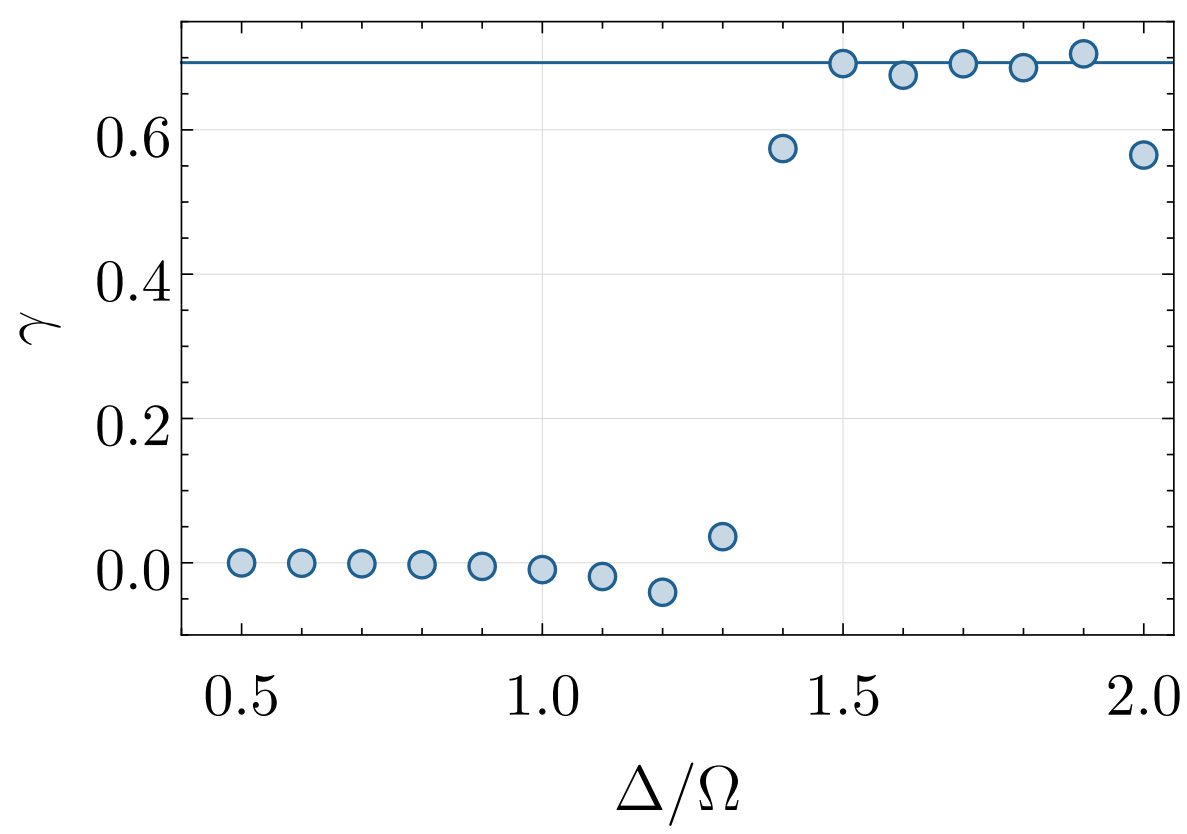

Figure 1: Topological entanglement entropy γ as a function of the detuning Δ/Ω for the model studied in this work. The saturation to $\gamma \simeq \ln 2$ over a broad parameter window is consistent with robust $\mathbb{Z}_2$ topological order.

6. *On a theoretical level, I wonder if comparing structure factors is really a useful quantity to identify the QSL. These are notoriously hard to measure in Rydberg atom arrays. Are there any other, more direct observables? Could one use projective measurements of some string order parameters as in Ref. 26, or some quench spectroscopy method (that provides access to the one-time correlation function)?*

   Response: We thank the referee for this very useful suggestion. We agree that structure factors are not the only—and in some settings not the most direct—diagnostic of a QSL, and that a wider set of observables should be mentioned. More direct probes include nonlocal string and loop operators, which can be measured projectively and were indeed used in the Rydberg spin-liquid experiment of Ref. [26] to characterize the topological phase. In our setting, such observables would provide a particularly natural probe of the $\mathbb{Z}_2$ QSL order, and could be used to distinguish QSL states from alternative topologically trivial phases.

   At the same time, the dynamical structure factor (DSF) remains a valuable object, as it encodes detailed information about the quasiparticle spectrum. Although the DSF can be experimentally challenging to access directly, it contains essentially the same spectral content as a variety of other dynamical response functions that can be probed using existing techniques.

   As the referee notes, quench spectroscopy is a particularly promising avenue: by evolving the system

after a sudden quench and measuring the resulting time-dependent correlations, one gains access to unequal-time correlation functions whose Fourier transforms yield spectral information analogous to the DSF. This approach has already been demonstrated in dipolar Rydberg XY systems in tweezer arrays [Chen *et al.*, *Science* 389, 483 (2025)]. More generally, several complementary experimental approaches can be used to access such dynamical or nonlocal observables:

- *Bragg spectroscopy.* In ultracold-atom systems, the DSF $\mathcal{S}(\mathbf{q}, \omega)$ can be measured by scattering far-detuned laser light off the atomic ensemble, directly probing density–density correlations in momentum and frequency space [Birkl *et al.*, *Phys. Rev. Lett.* 75, 2823 (1995)].
- *Phase-contrast (weak-measurement) imaging.* Homodyne weak-measurement schemes provide a minimally invasive probe of site-resolved densities. Two such measurements separated by a time delay yield unequal-time density correlations from the ensemble-averaged cross-correlations of the measurement outcomes [E Altuntaş *et al.*, *Quantum Sci. Technol.* 10 035045 (2025)].
- *Partial-transfer imaging.* This technique allows for repeated measurements of the same atomic ensemble with minimal disturbance, enabling time-resolved measurements of the dynamics and correlation functions [Seroka *et al.*, *Optics Express* 27, 36611 (2019)].
- *Randomized-measurement (classical-shadow) tomography.* Randomized-measurement protocols allow one to reconstruct an efficient classical representation ("shadow") of the quantum state, from which a wide range of observables—including correlation functions and entanglement measures—can be estimated with favorable scaling [Teng *et al.*, *PRX Quantum* 6, 040303 (2025)].

We therefore believe that, while challenging, measurements of quantities containing the same spectral information as the DSF are, in principle, accessible with current-generation experiments. We have added a brief remark outlining these possibilities in the revised manuscript.

In summary, we thank the referee for carefully reading our manuscript and making a considerable number of insightful comments. We believe that we have addressed all the issues raised, and that the incorporation of these changes has improved the quality of the paper to merit publication in *Nature Communications.*

Response to Referee 2

In this manuscript, Maity et al. presents a systematic classification of possible $\mathbb{Z}_2$ quantum spin liquid (QSL) states on the ruby lattice using fermionic parton mean-field theory and projective symmetry group (PSG) analysis. The authors further compute static and dynamical structure factors for all candidate ansatze, and identify a promising state consistent with their DMRG results.

Response: We thank the referee for a careful reading of the paper and for a concise and accurate summary of our work.

In my opinion, this is a important and innovative work. The manuscript is well-structured and and offers a valuable theory for understanding the QSLs in Rydberg atom arrays. I believe it is suitable for publication in Nature Communications after addressing the points outlined below.

Response: We thank the referee for their positive assessment of the manuscript. We address the detailed questions point-by-point below.

Major:

1. *The role of gauge fluctuations and their negligible effect on observable quantities could be explained more intuitively for a broader readership.*

   Response: We thank the referee for this suggestion and agree that this point deserves a more physical and accessible explanation.

   In the fermionic parton construction, the mean-field *Ansatz* is defined in an enlarged Hilbert space, and the associated gauge redundancy reflects the fact that different parton configurations can represent the same physical spin state. Gutzwiller projection enforces the local single-occupancy constraint and thereby restores the physical Hilbert space. In this sense, gauge fluctuations are not an additional microscopic ingredient introduced by hand; they are tied to the local constraint inherent in the parton representation.

   For the present work, the essential point is that the candidate state is a *fully gapped* $\mathbb{Z}_2$ spin liquid. In PSG language, the invariant gauge group is reduced from a continuous group to the discrete group $\mathbb{Z}_2$. Accordingly, there is no gapless emergent gauge boson at low energies—the gauge sector is itself gapped.

   This has an immediate consequence for observables. Since gauge fluctuations do not furnish an additional soft low-energy mode, they are not expected to qualitatively alter the long-distance physics, and their effects remain short-ranged. Moreover, because the spinon sector is also gapped, the low-energy theory is not subject to singular infrared corrections. Beyond-mean-field effects may renormalize nonuniversal quantities such as the overall spectral weight, linewidths, or short-distance amplitudes, but they are not expected to alter the qualitative momentum-space fingerprints that we use to distinguish the candidate *Ansätze*. We have revised the manuscript to make this physical point more explicit and accessible to a general readership.

2. *The comparison of SSF/DSF is good. Maybe the authors can discuss whether other observables (e.g., entanglement entropy, topological excitation spectra) could further distinguish between Ansatz states.*

   Response: This is an excellent suggestion. In principle, additional observables such as entanglement measures or excitation spectra could provide further information. However, in the present setting

their practical applicability is limited. The topological entanglement entropy is well defined (and yields a universal diagnostic of topological order) only for fully gapped phases; since not all *Ansätze* considered here are necessarily gapped, it cannot be used as a universal diagnostic across our entire classification. It can, however, be used to corroborate the $\mathbb{Z}_2$ nature of the specific candidate state, as is indeed confirmed by the DMRG measurement of the topological entanglement entropy (see our response to Referee 1, point 5).

A detailed analysis of topological excitation spectra, on the other hand, would require explicit access to the e and m quasiparticle sectors, which lies beyond the fermionic mean-field framework employed in this work (as discussed in our response to Referee 1, point 3). In contrast, the SSF and DSF are directly accessible observables that can be computed consistently for all *Ansätze* considered, making them natural diagnostics for a systematic comparison. We therefore focus on SSF and DSF as practical tools to distinguish between different PSG states, while leaving the study of complementary observables—in particular those targeting the e and m sectors—as an interesting direction for future work. We have added a brief remark to this effect in the revised manuscript.

3. *It would be good to briefly comment on whether Gutzwiller projection could qualitatively alter the structure factor predictions.*

   Response: We thank the referee for raising this important point. In general, Gutzwiller projection can modify structure-factor predictions, and in some cases the modification can be substantial; one should therefore not interpret mean-field structure factors as quantitatively exact.

   That said, the situation here is comparatively controlled. The candidate identified in the present work is a *fully gapped* $\mathbb{Z}_2$ spin liquid. Because the invariant gauge group is discrete, there is no gapless gauge mode, and because the spinons are also gapped, Gutzwiller projection is not expected to generate new infrared structure absent at the mean-field level. Its principal role is to enforce the local single-occupancy constraint exactly, while its quantitative effect is expected primarily in short-range correlations and in the redistribution of spectral weight.

   For this reason, although Gutzwiller projection need not leave the structure factor numerically unchanged, we do expect the *qualitative* momentum-space pattern to remain robust. In particular, the location of the dominant maxima, the broad distribution of spectral weight, and the presence or absence of characteristic features tied to a given PSG *Ansatz* should not be qualitatively altered by projection. Projection may change the contrast of these features and renormalize their intensity, but it is not expected to transform the fingerprint of one gapped $\mathbb{Z}_2$ *Ansatz* into that of another.

   We have added a brief remark in the manuscript to clarify both the usefulness and the limitations of the mean-field structure-factor comparison in this context.

4. *It would be helpful if you provide typical experimental parameter ranges (Ω, Δ, V_0) in the main text to enhance practical relevance.*

   Response: We thank the referee for this helpful suggestion. We have now included the relevant parameters in the revised main text. For our DMRG simulations, we use $\Delta/\Omega = 1.7$ and $V_{ij}/\Omega = 50$ for all pairs up to third-nearest neighbors, with the van der Waals interaction truncated beyond this range. These values are representative of the parameter regime relevant to Rydberg-array experiments: in the experiment of Ref. [26], for instance, $\Omega/(2\pi)$ is of the order of a few MHz and the detuning-to-Rabi-frequency ratio is of order $\Delta/\Omega \sim 1$–4, with V_0/Ω of several tens for nearest-neighbor pairs at the lattice spacings employed. Our choice places the system robustly inside the $\mathbb{Z}_2$ QSL phase previously identified for this Hamiltonian (see Ref. [27]), rather than attempting a quantitative fit to any single experimental realization.

Minor:

1. *The authors should provide details of the DMRG simulations, e.g., the lattice/geometry used in DMRG, the bond dimension, the converged energy, etc.*

   Response: We thank the referee for this suggestion and have added the corresponding DMRG details to the manuscript. In brief, the DMRG calculations were performed on a 288-site ruby lattice placed on a finite cylindrical geometry that is commensurate with the ruby unit cell. We retained bond dimensions sufficient to ensure that the truncation error was small (of order 10^{-6} or lower) and that the variational ground-state energy was converged to within the precision required for the observables reported in Fig. 2. Our DMRG results are in good agreement with those of the closely related kagome-lattice dimer-model study of Ref. [60], which provides an independent consistency check on the identification of the $\mathbb{Z}_2$ QSL phase.

In summary, we thank the referee for carefully reading our manuscript and making a considerable number of insightful comments. We believe that we have addressed all the issues raised, and that the incorporation of these changes has improved the quality of the paper to merit publication in *Nature Communications*.

Response to Referee 3

In this paper, the authors employ the projective symmetry group (PSG) framework to characterize symmetry fractionalization patterns in a topologically ordered $\mathbb{Z}_2$ quantum spin liquid (QSL) realized in a Rydberg atom array on the ruby lattice. They identify 64 $\mathbb{Z}_2$ projective extensions of the symmetry group, among which 50 are claimed to be realizable with a $\mathbb{Z}_2$ invariant gauge group (IGG). The authors further argue that these 50 possibilities are experimentally relevant and outline a concrete route for realizing them in the laboratory.

Response: We thank the referee for a careful reading of the paper and for a concise and accurate summary of our work.

This analysis is certainly interesting and physically well motivated. Personally, what I find most compelling are the static and dynamical structure factors presented in the paper, which provide clear signatures that can be probed both numerically and experimentally. I think it is overall a nice paper with significant scientific importance, and I would like to recommend its publication should the authors address my comments properly.

Response: We thank the referee for their positive assessment of the manuscript. We address the detailed questions point-by-point below.

The authors state that the PSG calculation must be carried out up to third-nearest-neighbor (3NN) bonds. Is this strictly necessary? What changes if one truncates the analysis at second-nearest neighbors (2NN), or alternatively extends it to fourth-nearest neighbors (4NN)? I suspect that the geometry of the ruby lattice imposes a natural cutoff, but this is not entirely clear from the current presentation.

Response: We thank the referee for pointing out the need for clarification here. The restriction of the PSG analysis to bonds up to third-nearest neighbors is not strictly necessary; it is adopted primarily for physical and computational convenience.

On the Rydberg platform, the effective interaction decays rapidly with distance as $V_{ij} \sim 1/r_{ij}^6$. This strong decay provides a natural physical justification for truncating the mean-field *Ansatz* after a few inter-site spacings; more distant bonds give only quantitatively small corrections and do not qualitatively affect the structure of the spin-liquid phases. In addition, for the $\mathbb{Z}_2$ spin-liquid phase on the ruby-lattice array, up to 3NN atoms sit within the Rydberg blockade radius, so 3NN captures the range of microscopic interactions that most directly participates in the QSL's formation.

Truncating the analysis at 2NN would further reduce the number of allowed mean-field parameters but would not change the underlying PSG constraints or symmetry classification; it would merely restrict the variational space of *Ansätze* associated with each PSG class. Extending the analysis to 4NN or beyond would increase the number of allowed parameters but is not expected to introduce new symmetry-distinct PSG classes—only additional symmetry-allowed amplitudes within the same classes. We have added a clarifying remark to this effect in the revised manuscript.

Much of the classic PSG literature focuses on systems with strictly local interactions. In the present setup, the interaction V is long-ranged. Could the authors clarify whether, and if so how, the long-range nature of V plays a role or not in the PSG classification?

Response: The PSG classification itself is determined solely by symmetry and does not depend on the range of the microscopic interactions. The long-range nature of the Rydberg interaction V_{ij} therefore does

not modify the classification but does enlarge the set of bonds on which symmetry-allowed mean-field amplitudes can be placed. Allowing longer-range interactions thus simply permits additional symmetry-allowed mean-field parameters within each PSG class. These extra variational degrees of freedom can improve the quantitative agreement of observables (e.g., structure factors) with the microscopic ground state, but they do not introduce new symmetry-distinct PSG classes. We have added a brief clarifying remark on this point in the revised manuscript.

I believe that the diagrams for the static and dynamical structure factors presented in the paper are calculated from the mean-field Ansatz. While this is a natural assumption, the strongly coupling nature of the system may have significant impact on the results. I wonder what is the authors' perspective on how the effect beyond mean-field theory may distort these diagrams or not.

Response: We agree that effects beyond mean-field theory can quantitatively affect the SSF and DSF, and we now discuss this point more carefully in the revised manuscript. There are two main sources of such corrections. First, gauge fluctuations about the mean-field saddle: in a fully gapped $\mathbb{Z}_2$ spin liquid such as the candidate identified here, the invariant gauge group is discrete and the gauge sector is itself gapped, so the gauge field mediates only short-ranged interactions and is not expected to qualitatively alter the long-distance momentum-space fingerprints (a more detailed discussion is given in our response to Referee 2, point 1). Second, Gutzwiller projection enforces the exact single-occupancy constraint; it is expected to improve quantitative agreement and to redistribute spectral weight, but—for a gapped $\mathbb{Z}_2$ *Ansatz*—is not expected to transform the qualitative fingerprint of one *Ansatz* into that of another (see our response to Referee 2, point 3). A systematic treatment of both effects is beyond the scope of the present work and is left for future studies.

Also, I would like to draw the authors' attention to several related works. In https://arxiv.org/abs/1507.07621*, the authors carry out a PSG classification for the same symmetry group and also obtain 64 solutions. It would be helpful if the present manuscript cited this work and commented on whether the agreement in the number of PSG solutions is coincidental or reflects a deeper structural reason. Furthermore, as emphasized in* https://arxiv.org/abs/1410.4540*, identifying symmetry fractionalization solely through its action on the e particles (as is done in standard PSG constructions) is generally insufficient. Some discussion of this point in the context of the present work would strengthen the manuscript. Relatedly, the more recent work* https://arxiv.org/abs/2309.15118 *provides a systematic approach to diagnosing symmetry fractionalization of the m particles; it would be interesting to hear the authors' perspective on the relation between these results and the PSG calculations presented in this manuscript.*

Response: We thank the referee for bringing these works to our attention. We have cited all three in the revised manuscript and comment on them in turn.

(i) *arXiv:1507.07621.* This work carries out a Schwinger-boson PSG classification on the *square* lattice. As the square lattice has a different space group from the ruby lattice, any coincidence in the number of projective extensions arises from the underlying group cohomology of the symmetry group used in the classification rather than from a direct structural correspondence between the two lattice problems. We therefore regard the numerical coincidence of "64 solutions" as just that—a coincidence—rather than evidence of a deeper structural relation. We have added a citation and a brief comment to this effect.

(ii) *arXiv:1410.4540.* We fully agree with the referee (and with the cited work) that a complete characterization of symmetry-enriched topological order requires tracking symmetry fractionalization on *all* anyonic sectors, not just the e particles. The fermionic PSG analysis we present here directly characterizes the symmetry fractionalization of the ε particles; as discussed in our response to Referee 1, point 3, a complete characterization of the Rydberg $\mathbb{Z}_2$ QSL would further require bosonic PSG analyses on the

kagome (e) and dice (m) lattices. We have added a remark in the revised manuscript acknowledging this and citing the referee's suggested reference.

(iii) *arXiv:2309.15118.* This work provides a powerful and systematic framework to diagnose the symmetry fractionalization of the m particle directly at the level of the symmetry group, without relying on a specific parton construction. Our PSG analysis, by contrast, is formulated within a fermionic parton mean-field framework and characterizes the ε particle. While both approaches ultimately address symmetry-enriched topological order, they focus on complementary quasiparticle sectors and employ conceptually different methodologies. In the Rydberg QSL considered here, the m particles reside on the dice lattice, distinct from the ruby lattice on which the fermionic spinons live; a direct application of the vison-diagnosis framework of arXiv:2309.15118 would therefore require a separate, lattice-specific analysis on the dice lattice.

We view the results of arXiv:2309.15118 as highly complementary to our PSG classification. Establishing a detailed correspondence between the vison symmetry fractionalization diagnosed in that framework and the fermionic PSG classes identified here is an interesting and important direction for future work, and we have added a remark to this effect in the revised manuscript.

In summary, we thank the referee for carefully reading our manuscript and making a considerable number of insightful comments. We believe that we have addressed all the issues raised, and that the incorporation of these changes has improved the quality of the paper to merit publication in *Nature Communications*.

Reviewer #1

Comment: The authors have addressed my comments and questions in a satisfactory manner. In particular, they have clarified relations to their earlier works which also highlights the significance of their new results. I therefore recommend the paper for publication in Nat. Comms.

Response: We thank the reviewer for their positive assessment of the revised manuscript and for recommending publication. We are grateful for the reviewer's earlier comments, which helped us clarify the relation between the present work and our previous studies, and thereby sharpen the presentation of the new results.

Comment on code availability: I could not find any openly available code or data.

Response: We thank the reviewer for pointing this out. We have now made the relevant code and numerical data openly available through the Zenodo repository:

https://doi.org/10.5281/zenodo.20696295

The revised manuscript includes this DOI explicitly in the Data Availability and Code Availability statements. The numerical data underlying the figures, including the DMRG ground-state structure factors and the mean-field structure factors for the $\mathbb{Z}_2$ Ansätze, have been deposited there. The custom code used for the projective symmetry group classification, construction of the mean-field Ansätze, and calculation of static and dynamical structure factors has also been deposited at the same repository. We believe this addresses the reviewer's concern.

Reviewer #2

Comment: The reviewer states that the resubmission addresses all concerns, including the distinction from previous work, the inclusion of triplet channels, the justification of mean-field

theory in the gapped $\mathbb{Z}_2$ QSL, the inclusion of topological entanglement entropy data in the reply, and the listing of relevant experimental parameters. The reviewer recommends publication.

Response: We sincerely thank the reviewer for their careful reading of the revised manuscript and for their positive recommendation. We are pleased that the revisions clarified the distinction between the present work and earlier studies, especially the role of the longitudinal triplet channels relevant to the Rydberg setting. We are also grateful that the reviewer found the discussion of the mean-field approach, the topological entanglement entropy, and the experimentally relevant parameters useful.

Reviewer #3

Comment: The reviewer thanks the authors for the careful reply and states that the authors have properly addressed all questions. The reviewer recommends publication in *Nature Communications*.

Response: We thank the reviewer for their careful evaluation of our revised manuscript and for recommending publication. We are grateful for the reviewer's earlier questions, which helped us improve the clarity and completeness of the presentation.

We hope that the revised manuscript and the accompanying files now satisfy all requirements for publication in *Nature Communications*.
